# Anthropoid primate–specific retroviral element THE1B controls expression of *CRH* in placenta and alters gestation length

**DOI:** 10.1371/journal.pbio.2006337

**Published:** 2018-09-19

**Authors:** Caitlin E. Dunn-Fletcher, Lisa M. Muglia, Mihaela Pavlicev, Gernot Wolf, Ming-An Sun, Yueh-Chiang Hu, Elizabeth Huffman, Shivani Tumukuntala, Katri Thiele, Amrita Mukherjee, Sandra Zoubovsky, Xuzhe Zhang, Kayleigh A. Swaggart, Katherine Y. Bezold Lamm, Helen Jones, Todd S. Macfarlan, Louis J. Muglia

**Affiliations:** 1 Division of Human Genetics, Center for Prevention of Preterm Birth, Perinatal Institute, Cincinnati Children’s Hospital Medical Center, Department of Pediatrics, University of Cincinnati College of Medicine, Cincinnati, Ohio, United States of America; 2 The Eunice Kennedy Shriver National Institute of Child Health and Human Development, The National Institutes of Health, Bethesda, Maryland, United States of America; 3 Division of Developmental Biology, Cincinnati Children’s Hospital Medical Center, Department of Pediatrics, University of Cincinnati College of Medicine, Cincinnati, Ohio, United States of America; 4 Division of Pediatric Surgery, Cincinnati Children’s Hospital Medical Center, Department of Surgery, University of Cincinnati College of Medicine, Cincinnati, Ohio, United States of America; University of Cambridge School of Clinical Medicine, United Kingdom of Great Britain and Northern Ireland

## Abstract

Pregnancy and parturition are intricately regulated to ensure successful reproductive outcomes. However, the factors that control gestational length in humans and other anthropoid primates remain poorly defined. Here, we show the endogenous retroviral long terminal repeat transposon-like human element 1B (THE1B) selectively controls placental expression of corticotropin-releasing hormone (*CRH*) that, in turn, influences gestational length and birth timing. Placental expression of *CRH* and subsequently prolonged gestational length were found in two independent strains of transgenic mice carrying a 180-kb human bacterial artificial chromosome (BAC) DNA that contained the full length of *CRH* and extended flanking regions, including THE1B. Restricted deletion of THE1B silenced placental *CRH* expression and normalized birth timing in these transgenic lines. Furthermore, we revealed an interaction at the 5′ insertion site of THE1B with distal-less homeobox 3 (DLX3), a transcription factor expressed in placenta. Together, these findings suggest that retroviral insertion of THE1B into the anthropoid primate genome may have initiated expression of *CRH* in placental syncytiotrophoblasts via DLX3 and that this placental CRH is sufficient to alter the timing of birth.

## Author summary

The proper timing of delivery is critical during pregnancy; if too early or too late, the baby will be at risk of serious health problems and even death. Corticotropin-releasing hormone (CRH) is a protein that can be detected in maternal blood, and its concentration correlates with the timing of birth. In humans and other anthropoid primates, CRH is made by the placenta, whereas in other mammals, it is produced in a specialized region of the brain. To understand the regulation and evolution of this key protein, we inserted the human *CRH* gene and nearby regions into the mouse genome, which resulted in human *CRH* expression in the mouse placenta. Mouse litters that make CRH in their placentas are born later than control mice, showing that CRH can directly affect birth timing. Using our mouse model, we then selectively deleted a remnant of an ancient retrovirus that is normally found in the DNA of anthropoid primates and demonstrated that this specific region controls expression of *CRH* in the placenta. Deletion of this region also restored normal birth timing in the mice by eliminating CRH production from the placenta. We propose that retroviral regulation of *CRH* in the placenta may be a mechanism of controlling birth timing in humans and other anthropoid primates.

## Introduction

The complex process of completing gestation and initiating parturition must be tightly controlled to prevent the dangerous consequences of preterm or postterm birth to the mother and offspring. In humans, corticotropin-releasing hormone (CRH) production by the placenta increases exponentially with gestational age, predicting the onset of parturition [1]. This exponential increase in placental CRH production has been observed earlier in the pregnancy when that pregnancy was destined to end in preterm birth and later in the pregnancy for postterm birth, indicating that placental CRH may play a role in the timing and onset of labor [1–4].

The peptide hormone CRH has a well-characterized role in the hypothalamic-pituitary-adrenal axis that is highly conserved in vertebrates [5]. However, its expression in the placenta is observed only in anthropoid primate species, consistent with evolutionary changes in placental gene regulation [6–14]. One major driver of evolutionary diversity in gene regulation is the class of mobile DNA sequences known as transposable elements [15, 16]. Recent advances in genome sequencing, high-throughput screening for active chromatin, and computational resources facilitating comparative genomic analysis have made it possible to identify lineage-specific DNA sequences with signs of regulatory activity, many of which are derived from transposable elements [17–21].

In a recent study, species-specific enhancers active in early placental development were found to be highly enriched for long terminal repeats (LTRs), a subset of transposable elements derived from retroviruses [22]. LTR regions of retroviruses utilize available host factors to recruit transcription machinery and produce virus, and isolated LTRs have been reported to function as promoters or enhancers of the host’s genes [23–28]. To understand whether similar co-option of LTRs into the host’s function has occurred in placental regulation of *CRH* expression, we examined the vicinity of the *CRH* gene for elements that are conserved in anthropoid primates but not present in species without placental *CRH* expression. We identified a retroviral LTR element of the transposon-like human element 1B (THE1B) family, which invaded the anthropoid primate genome approximately 50 million years ago [29]. We hypothesized that the introduction of this retroviral LTR element into the genome of anthropoid primate common ancestors initiated expression of *CRH* in placental tissue. Here, we tested this hypothesis by introducing the human *CRH* locus into transgenic mouse lines and selectively editing regions of a nearby THE1B LTR element to control the expression of human *CRH* in mouse placental tissue. We show that this LTR element interacts with distal-less homeobox 3 (DLX3), a transcription factor (TF) required for placental development [30]. Transgenic mice expressing human *CRH* in their placentas exhibited significant changes in their length of gestation, suggesting that placental *CRH* expression is a potential mechanism of controlling the timing of birth that is unique to anthropoid primates.

## Results

### *CRH* expression and retroviral element THE1B presence are concordant in anthropoid primate placenta

**Fig 1:**
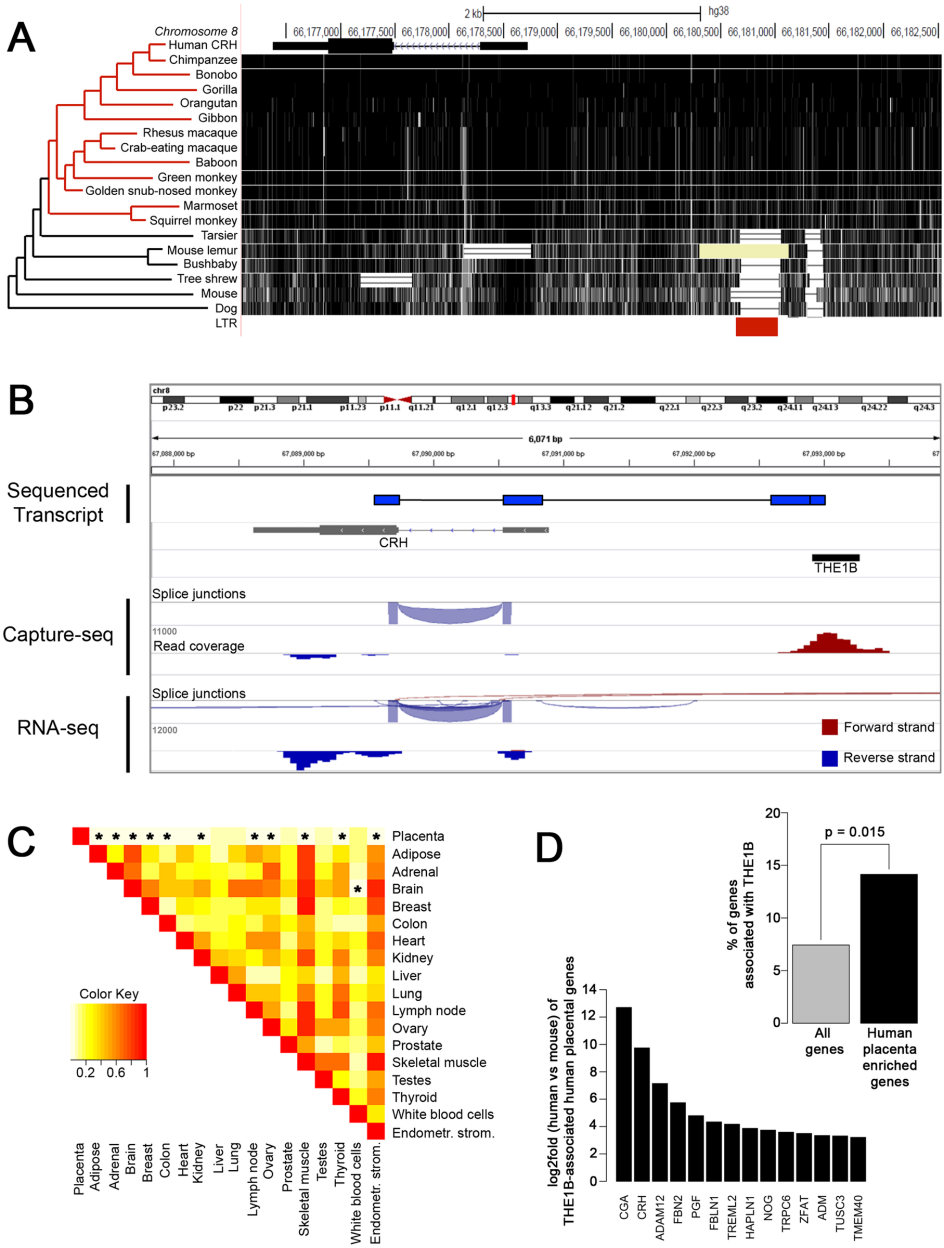
THE1B is a candidate enhancer for placenta-specific regulation of *CRH* and other genes. (A) Comparative genomic alignment of 16 primates and 3 nonprimate mammals (UCSC Genome Browser, hg38, accessed June 14, 2017). LTR element THE1B (red bar) is present in anthropoid primates (red lines of phylogenetic tree) and absent in prosimians and nonprimate mammals. (B) Alignment of PCR-amplified transcript (Sequenced Transcript) and reads from human placental transcriptome precapture (RNA-seq) and postcapture (Capture-seq) for THE1B-containing transcripts. Sequenced fusion transcript found in human and rhesus macaque joins THE1B to *CRH* exon 1. This transcript is not found in pre- or postcapture RNA-seq; however, THE1B is transcribed. Raw data can be found at GEO accession number GSE118289. (C) Heat map of coregulation of THE1B-associated genes in placenta relative to other tissues. THE1B-associated genes are less coregulated, and thus more divergent in expression, between placenta and other tissues when compared to a group of randomly selected genes. Numerical values and their corresponding colors represent the odds ratio of observing the effect of relative coexpression between tissue pairs, generated by nonparametric tests separately between each pair of tissues as described by Pavlicev and colleagues [33]. (D) Upper right, higher percentage of human placenta–enriched genes, compared with all genes, are associated with THE1B. (*P* = 0.015 by binomial test.) Lower left, the log2fold gene expression between human and mouse of THE1B-associated human placenta–enriched genes. Raw data can be found at GEO accessions GSE118289 and GSE43520 (human placenta), GSE43520 (mouse placenta), and GSE30611 (other human tissues). Numerical data can be found in S1 Data. CRH, corticotropin-releasing hormone; Endometr. strom., endometrial stromal cells; GEO, Gene Expression Omnibus; LTR, long terminal repeat; RNA-seq, RNA sequencing; THE1B, transposon-like human element 1B; UCSC, University of California, Santa Cruz.

Because *CRH* is expressed in placenta of anthropoid primate species only, we hypothesized that any potential regulatory DNA sequence would be conserved only in the anthropoid primate lineage. We compared the genomes of 12 anthropoid primates, 3 prosimians, and 3 nonprimate mammals in the region of *CRH* and identified an endogenous retroviral LTR of the THE1B type that correlated with placental *CRH* expression (Fig 1A). THE1B LTR elements have been shown to activate transcription under certain conditions [31, 32]. To determine activity of this THE1B element in anthropoid primate placenta, we utilized a PCR screening system to amplify transcripts containing both THE1B and the coding exon of *CRH* from placental tissue. Both human and rhesus macaque placentas expressed a detectable fusion transcript with an identical splice junction connecting sequence downstream of THE1B to noncoding exon 1 of *CRH* (Fig 1B). This novel splice junction is conserved in the primate species included in Fig 1A, suggesting a possible role for THE1B as a regulatory element of *CRH* in the placenta (S1 Table). The THE1B-CRH fusion transcript was detectable by PCR screening but not detectable by RNA sequencing (RNA-seq) from term human placenta, indicating low abundance relative to total *CRH* expression (Fig 1B). To better detect potential THE1B-CRH fusion transcripts, we utilized a capture sequencing approach in which we first enriched for cDNAs containing THE1B elements using a library of biotinylated complementary RNA probes that could be pulled down with streptavidin beads prior to deep sequencing. Despite obtaining greater than 1,000-fold enrichment of THE1B-containing transcripts, we remained unable to detect any chimeric reads linking THE1B and *CRH*. In contrast, we found abundant reads transcribed in the antisense direction through the THE1B element (Fig 1B). Because these reads are unidirectional and within 2 kb of the *CRH* promoter region, it is likely that these reads were initiated from a bidirectional *CRH* promoter. In sum, the comparative genomics and RNA-seq data led us to hypothesize that the anthropoid-specific THE1B element serves as an enhancer, not an alternative promoter, of the *CRH* gene.

### THE1B LTRs may form a coordinated regulatory network in anthropoid primate placenta

Previous studies have implicated endogenous retroviral LTR elements as promoters and enhancers that drive placenta-specific gene expression [22, 33–38]. To examine the effect of THE1B LTRs on placental expression of genes, including *CRH*, we first defined a set of genes that had a THE1B element within 10 kb of the transcription start site. This set of THE1B-associated genes contained 2,311 gene–THE1B pairings, encompassing about 10% of THE1B elements in the human genome. We then compared the expression pattern of THE1B-associated genes across different tissues using a relative coexpression analysis as described by Pavlicev and colleagues [33]. Briefly, the analysis was run on all expressed genes and tested whether the THE1B-associated genes were equally correlated in their expression between a focal tissue and each of 17 other tissues than expected for a random size-matched subset of THE1B-unassociated genes. Consistently lower correlations in all tests involving a tissue of interest imply that some feature of THE1B-associated genes confers the particular expression status in that particular tissue, and in fact, the relative coexpression of THE1B-associated genes in tests involving placenta was significantly lower than for a random gene set (Fig 1C). An association between a THE1D element and placental expression of a single gene has been reported [39]; however, Pavlicev and colleagues found no change in relative coexpression of genes associated with other THE1 family members in placenta [33].

In a second analysis, we quantified gene expression from RNA-seq of human and mouse tissues to identify genes with human-specific, placenta-enriched gene expression. We then examined whether these differentially expressed genes were enriched for THE1B elements within 20 kb of their promoters relative to a control set of genes. Indeed, we found a significant association of THE1B elements with human placenta–enriched genes (Fig 1D). Taken together, these data show that genomic presence of THE1B is associated with differential expression of nearby genes in anthropoid primate placenta.

Enrichment of histone H3 lysine 27 acetylation (H3K27ac) or histone H3 lysine 4 monomethylation (H3K4me1), histone modifications associated with classical enhancers, was not detected at the THE1B upstream of *CRH* in chromatin immunoprecipitation sequencing (ChIP-seq) analyses performed on human placental tissue; however, other genomic THE1B elements were associated with these modifications (S1 Fig).

### THE1B-CRH transgenic mice express human *CRH* in placenta

**Fig 2:**
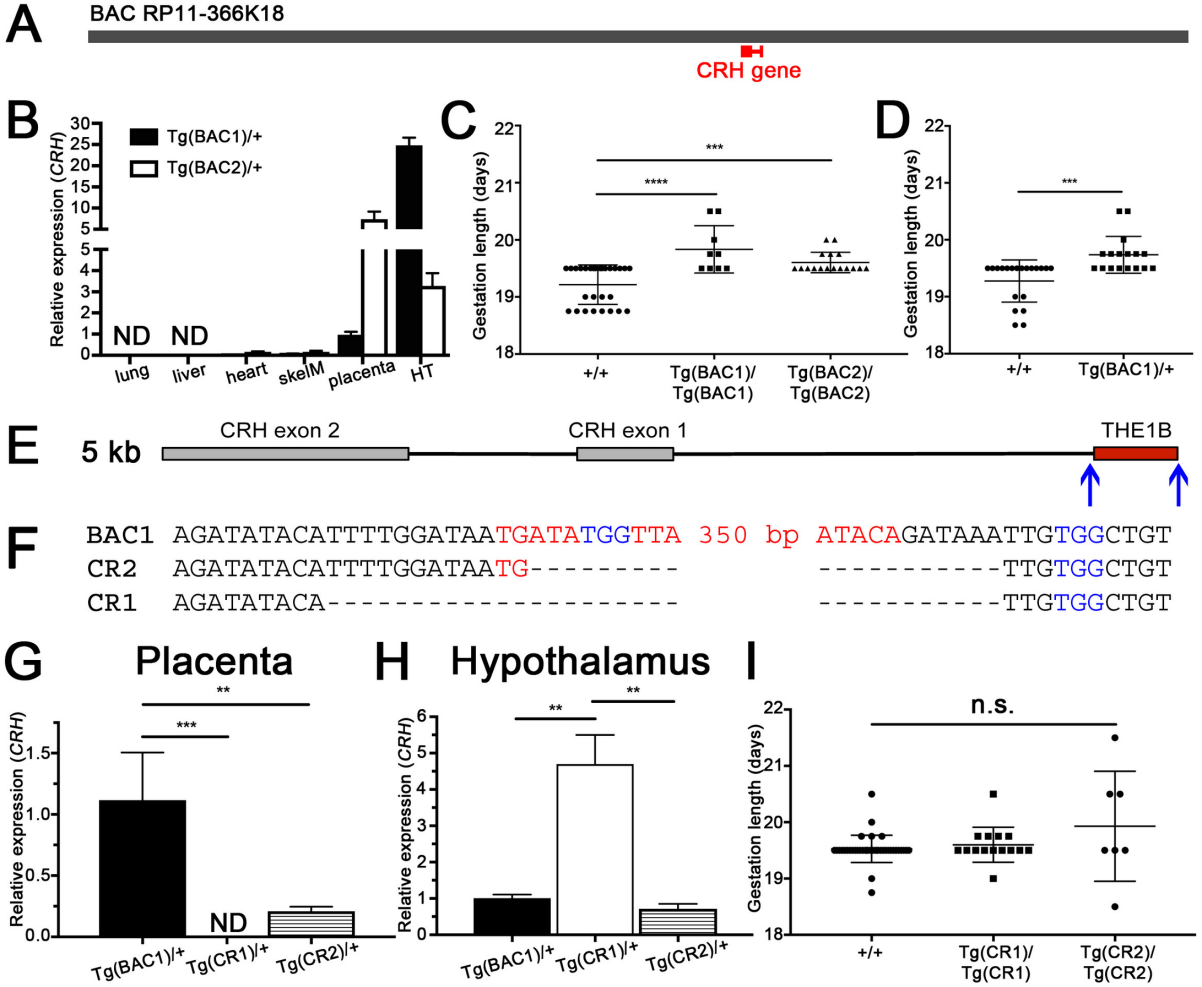
BAC transgenic mice exhibit placental *CRH* expression and delayed parturition, which are eliminated by THE1B deletion. (A) Schematic of human BAC clone RP11-366K18 (gray) with *CRH* shown to scale (red). (B) Expression of human *CRH* measured by qPCR in adult tissues and placenta. (*n* = 2–4 per group, *P* < 0.0001 by two-way ANOVA with tissue type as source of variation. Error bars indicate the standard error of the mean.) (C) Gestation length of litters homozygous for Tg(BAC1), homozygous for Tg(BAC2), or wild-type control. BAC transgenic litters have a significantly longer gestation length than wild-type control litters. (*n* = 28 +/+, *n* = 9 Tg[BAC1]/Tg[BAC1], *P* < 0.0001 by one-way ANOVA with post hoc Tukey’s multiple comparisons test. *n* = 17 Tg[BAC2]/Tg[BAC2], *P* = 0.0006 compared to +/+ by one-way ANOVA with post hoc Tukey’s multiple comparisons test. Error bars indicate standard deviation.) (D) Gestation length of litters hemizygous for Tg(BAC1) or wild-type control. Litters receiving Tg(BAC1) only from the father are born significantly later than wild-type control litters. (*n* = 20 +/+, *n* = 17 Tg[BAC1]/+, *P* = 0.0003 by unpaired two-tailed *t*-test. Error bars indicate the standard deviation.) (E) Schematic of 5-kb region containing *CRH* (gray) and THE1B (red). Blue arrows represent PAM sequences targeted by CRISPR/Cas9. (F) Sequenced deletions of the indicated transgenic founder animals compared to the Tg(BAC1) parent line. Red text, THE1B element. Blue text, PAM sequences indicated in (A). (G) Placental expression of human *CRH* at E18.5 measured by qPCR. (*n* = 3 Tg[BAC1]/+, *n* = 6 Tg[CR1]/+, *P* = 0.0006 by one-way ANOVA with post hoc Tukey’s multiple comparisons test. *n* = 5 Tg[CR2]/+, *P* = 0.0035 compared to Tg[BAC1]/+ by one-way ANOVA with post hoc Tukey’s multiple comparisons test. Error bars indicate the standard error of the mean.) (H) Hypothalamic expression of human *CRH* measured by qPCR. (*n* = 3 Tg[BAC1]/+, *n* = 4 Tg[CR1]/+, *P* = 0.0033 by one-way ANOVA with post hoc Tukey’s multiple comparisons test. *n* = 4 Tg[CR2]/+, *P* = 0.0013 compared to Tg[CR1]/+ by one-way ANOVA with post hoc Tukey’s multiple comparisons test. Error bars indicate the standard error of the mean.) (I) Gestation length of litters homozygous for Tg(CR1), homozygous for Tg(CR2), or wild-type control. The gestation length of THE1B-deleted transgenic litters is not significantly different from wild-type control litters. (*n* = 39 +/+, *n* = 15 Tg[CR1]/Tg[CR1], *n* = 7 Tg[CR2]/Tg[CR2], *P* = 0.0568 by one-way ANOVA. Error bars indicate standard deviation.) Numerical data for B, C, D, G, H, and I can be found in S1 Data. BAC, bacterial artificial chromosome; CRISPR/Cas9, clustered regularly interspaced short palindromic repeat/CRISPR-associated 9; E18.5, embryonic day 18.5; HT, hypothalamus; ND, not detected; ns, not significant; PAM, protospacer adjacent motif; qPCR, quantitative PCR; skelM, skeletal muscle; THE1B, transposon-like human element 1B.

In order to study the regulation of *CRH* in placenta, we created a novel mouse model by incorporating a bacterial artificial chromosome (BAC) with the human *CRH* gene and approximately 180 kb of flanking sequence, including the THE1B LTR, into the genome of FVB/N mice (Fig 2A). Random integration of this BAC resulted in two founder animals, denoted Tg(BAC1) and Tg(BAC2), which were bred independently to C57BL/6 mice and maintained as separate lines. We then tested for the presence of human *CRH* in adult tissues and determined that expression of *CRH* in Tg(BAC1)/+ and Tg(BAC2)/+ animals is remarkably specific to the hypothalamus and placenta at embryonic day 18.5 (E18.5) (Fig 2B). No human *CRH* was detected in any tissues of nontransgenic littermates.

### Placental expression of human *CRH* delays parturition in transgenic mice

As expression of *CRH* in human placenta is known to correlate with timing of parturition [1], we examined the timing of birth in Tg(BAC1) and Tg(BAC2) mouse lines. When compared with strain-matched control litters, Tg(BAC1)/Tg(BAC1) litters were born an average of 14.9 hours later (one-way ANOVA with Tukey post hoc, *P* < 0.0001; Fig 2C), and Tg(BAC2)/Tg(BAC2) litters were born an average of 9.3 hours later (one-way ANOVA with Tukey post hoc, *P* = 0.0006; Fig 2C), suggesting that placental expression of human *CRH* is sufficient to alter gestation length in mice. This effect of CRH appears to be independent of progesterone withdrawal, as no differences were detected in maternal serum progesterone or in uterine expression of the contractile-associated proteins oxytocin receptor (*Oxtr*), connexin-43 (*Gja1*), caveolin-1 (*Cav1*), cyclooxygenase 1 (COX-1; *Ptgs1*), or cyclooxygenase 2 (COX-2; *Ptgs2*) at E18.5 (S2 Fig). A significant decrease in prostaglandin F2α was noted in uterine tissue of Tg(BAC1)/Tg(BAC1) litters at E18.5 (S2 Fig).

To eliminate the possibility that this alteration of gestation length was a result of hypothalamic human *CRH* expression, we repeated these experiments with C57BL/6 (nontransgenic) mothers. Wild-type mice delivering Tg(BAC1)/+ litters completed gestation an average of 11.0 hours later than strain-matched control litters (unpaired two-tailed *t*-test, *P* = 0.0003; Fig 2D), associating the delayed parturition phenotype with the genotype of the fetal-derived placenta.

### Deletion of THE1B by CRISPR/Cas9 eliminates placental *CRH* expression

To determine if retroviral LTR THE1B is necessary for placental *CRH* expression, we used the clustered regularly interspaced short palindromic repeat/CRISPR-associated 9 (CRISPR/Cas9) system to specifically delete THE1B from the genome of Tg(BAC1) mice. We utilized two guide RNAs targeting the 5′ end of THE1B and the 3′ end immediately flanking the 366-bp THE1B sequence such that the THE1B element would be deleted by nonhomologous end joining of the surrounding sequence (Fig 2E). Microinjection of guide RNAs and *Cas9* mRNA into the cytoplasm of the zygotes generated by Tg(BAC1)/+ crosses resulted in two founder animals with deletions greater than 300 bp. Sanger sequencing of the deletions revealed that the larger deletion, termed Tg(CR1), completely lacked the THE1B element (Fig 2F). The other deletion, termed Tg(CR2), contained 12 bp at the 5′ end of THE1B that were deleted in Tg(CR1). This 12-bp sequence is comprised of 2 bp of THE1B sequence and 10 bp immediately upstream of the THE1B element (Fig 2F).

Next, we measured the mRNA expression of human *CRH* in placenta of Tg(CR1)/+ and Tg(CR2)/+ animals at E18.5. Complete deletion of THE1B in the Tg(CR1) line abolished placental expression of human *CRH* (one-way ANOVA with Tukey post hoc, *P* = 0.0006 compared to Tg[BAC1]; Fig 2G). The Tg(CR2) line retains 2 bp of the 5′ end of THE1B and subsequently expressed about 20% human *CRH* relative to the Tg(BAC1) parent line (one-way ANOVA with Tukey post hoc, *P* = 0.0035; Fig 2G).

We next examined the effect of THE1B deletion on the other primary site of *CRH* expression, the hypothalamus. As expected, deletion of THE1B did not abolish hypothalamic expression of human *CRH* in either transgenic line. Complete deletion of THE1B in the Tg(CR1) line appeared to increase expression of human *CRH* in the hypothalamus (one-way ANOVA with Tukey post hoc, *P* = 0.0033 compared to Tg[BAC1], *P* = 0.0013 compared to Tg[CR2]; Fig 2H). We detected compensatory down-regulation of mouse *Crh* in hypothalamus, resulting in no difference in serum corticosterone relative to nontransgenic mice (S3 Fig). We also examined the effect of THE1B deletion on the other protein-coding gene contained on the BAC, *TRIM55*, which is a ubiquitin E3-ligase expressed in striated muscle [40, 41]. *TRIM55* expression was detectable by RNA-seq in Tg(BAC1) placenta but not in Tg(CR1) placenta at E18.5, demonstrating that THE1B elements may control expression of multiple genes in a region (S4 Fig).

### Deletion of THE1B by CRISPR/Cas9 restores wild-type gestation length

To further interrogate the role of placental CRH in delayed onset of parturition, we examined the gestation length of Tg(CR1) and Tg(CR2) animals. The gestation length of litters homozygous for either Tg(CR1) or Tg(CR2) was not significantly different from strain-matched control litters (one-way ANOVA, *P* = 0.0568; Fig 2I), demonstrating that complete deletion of THE1B and subsequent lack of placental CRH rescued the extended-gestation phenotype of the Tg(BAC1) parent line. Tg(CR2)/Tg(CR2) litters had unusual variability in their gestation length, which could be due to the low level of expression of *CRH* from the residual 5′ insertion site of THE1B (Fig 2G). Despite this variability, Tg(CR2)/+ litters showed no differences in birth timing relative to control litters (S5 Fig).

### TF DLX3 interacts with THE1B 5′ insertion site

**Fig 3:**
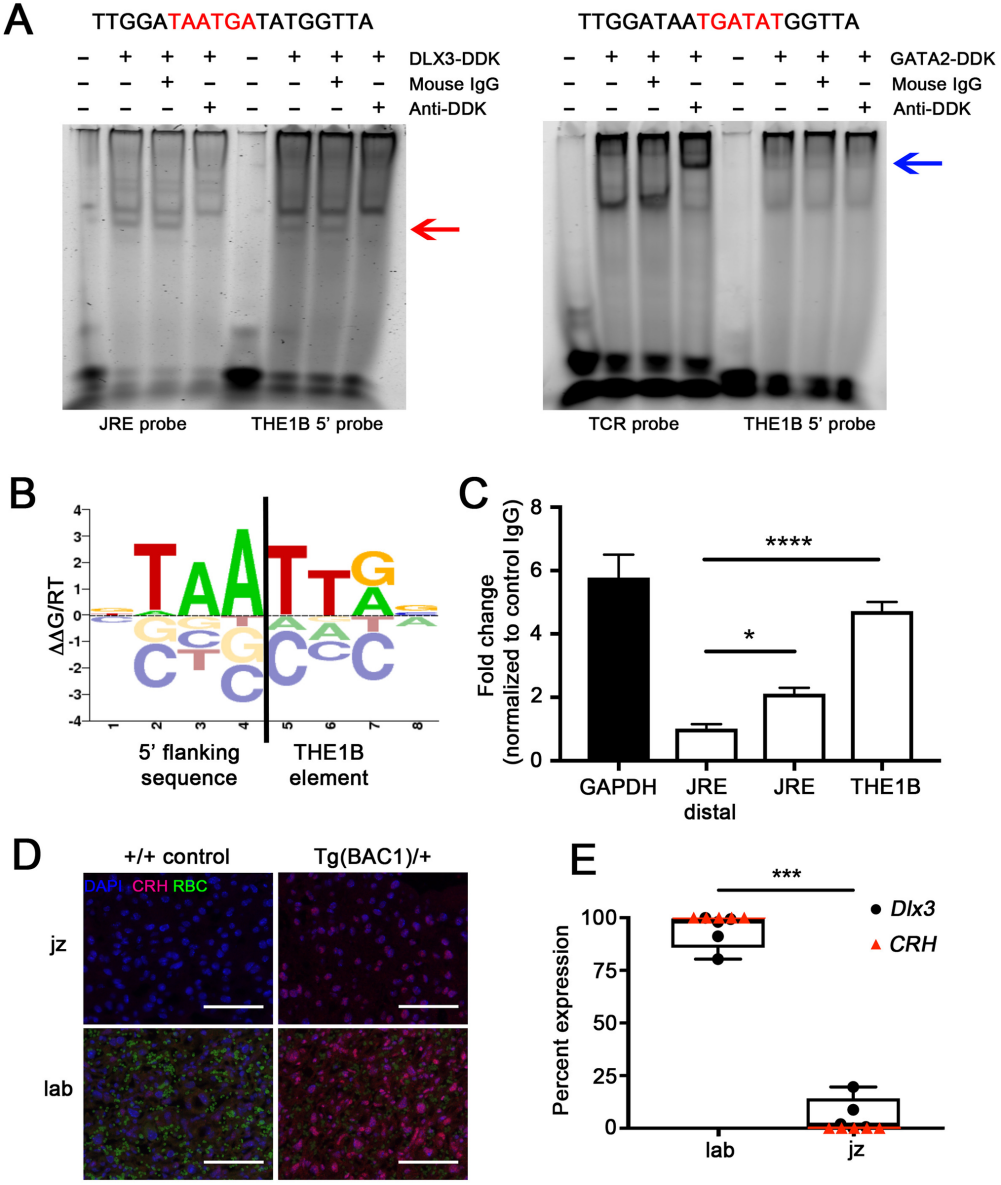
THE1B 5′ insertion site creates novel binding site for transcription factor DLX3. (A) Top left, sequence of 5′ insertion site of THE1B near *CRH*, predicted DLX3 binding site shown in red. Lower left, electrophoretic mobility shift assay demonstrating binding of DLX3-DDK to positive control (JRE probe) and the 5′ insertion site of THE1B (THE1B 5′ probe). The red arrow denotes the band formed by DLX3-DDK binding to labeled DNA probe; this binding is disrupted with the addition of anti-DDK but not isotype control mouse IgG. Top right, same sequence as left, predicted GATA2 binding site shown in red. Lower right, GATA2-DDK fails to bind to the 5′ insertion site of THE1B (THE1B 5′ probe) while binding positive control (TCR probe). Blue arrow denotes the supershifted band of anti-DDK, GATA2-DDK, and TCR probe. (B) DNA binding motif of transcription factor DLX3 [46]. This DLX3 binding site is formed by the insertion of THE1B (right of black bar) into the genome (left of black bar). (C) ChIP for DLX3 (black bars) and RNA polymerase II (white bar) with quantitative real-time PCR. DLX3 is significantly associated with the 5′ end of THE1B and positive control (JRE) in human term placenta. ChIP-qPCR data were normalized to the IgG control for each target and presented relative to negative control (JRE distal). (*n* = 3 for all, *P* < 0.0001 for THE1B and *P* = 0.0184 for JRE by one-way ANOVA with post hoc Dunnett’s multiple comparisons test relative to JRE distal. Error bars indicate the standard error of the mean.) (D) Immunohistochemical comparison of human CRH localization in junctional zone (“jz”) and labyrinth (“lab”) of Tg(BAC1)/+ and wild-type littermate control. Human CRH is predominantly localized to the labyrinth in Tg(BAC1) mouse placenta. Blue, nuclei with DAPI. Magenta pseudocolor, CRH (imaged on far red). Green, background fluorescence of RBCs. Scale bars, 100 μm. (E) Quantitative real-time PCR of *Dlx3* and *CRH* in Tg(BAC1)/+ mouse placentas separated by dissection into junctional zone (“jz”) and labyrinth (“lab”). *Dlx3* (black circle) is predominantly expressed in labyrinth, which is consistent with previous reports [47]. (*n* = 5 Tg(BAC1)/+, *P* = 0.0003 by paired two-tailed *t*-test. Box and whiskers indicate that all data points are displayed.) *CRH* (red triangle) is undetectable in junctional zone. Numerical data for C and E can be found in S1 Data. ChIP, chromatin immunoprecipitation; CRH, corticotropin-releasing hormone; DDK, aspartic acid–aspartic acid–lysine; DLX3, distal-less homeobox 3; GAPDH, glyceraldehyde 3-phosphate dehydrogenase; IgG, immunoglobulin G; JRE, human glycoprotein hormone α-subunit junctional regulatory element; qPCR, quantitative PCR; RBC, red blood cell; TCR, T cell receptor; THE1B, transposon-like human element 1B.

Transposable elements, especially those arising from retroviral LTRs, can activate gene expression by recruiting binding of available TFs [18, 22]. To determine the pool of TFs present in anthropoid primate placenta when *CRH* is known to be highly expressed, we analyzed placental transcriptomes generated by RNA-seq from two human and two rhesus macaque placentas near term. We identified a set of 90 TFs expressed at 10 transcripts per million (TPM) or greater in all samples, which we defined as moderately expressed TFs, and proceeded to examine potential binding of these TFs to the THE1B sequence with the prediction tool CisBP [42]. Two families of TFs known to contribute to placental trophoblast differentiation, GATA [43] and DLX [44], were predicted by CisBP to interact with the 5′ end of the THE1B sequence that was retained in Tg(CR2) but deleted in Tg(CR1). When tested by gel shift assay, DLX3, but not GATA2, was able to bind the 5′ end of THE1B (Fig 3A). This DLX3 binding site is located at the junction of the THE1B element and the surrounding DNA (Fig 3B).

We then performed ChIP with anti-DLX3 antibody in human term placental tissue and quantified by real-time PCR to determine the occupancy of this DLX3 binding site in vivo. DLX3 was significantly associated with the 5′ end of THE1B and with a previously described placental regulatory element [45], when compared to a negative control region (one-way ANOVA with Dunnett’s post hoc, *P* < 0.0001 for THE1B and *P* = 0.0184 for human glycoprotein hormone α-subunit junctional regulatory element [JRE] relative to JRE distal; Fig 3C).

### DLX3 binding to THE1B may drive *CRH* expression in syncytiotrophoblasts

In human placental tissue, the syncytium is the site of *CRH* expression and secretion into maternal and fetal circulations [48, 49]. We demonstrated localization of human CRH to the labyrinth of the Tg(BAC1)/+ mouse placenta, which contains mouse syncytiotrophoblasts (Fig 3D). We also confirmed by quantitative PCR (qPCR) that expression of human *CRH* was absent in the junctional zone (Fig 3E). Despite differences in placental morphology between human and mouse, DLX3 is produced in both human [50] and mouse [47] syncytiotrophoblasts. In Tg(BAC1)/+ placenta, 80%–99% of total *Dlx3* expression was localized to the labyrinth tissue (*n* = 5 Tg[BAC1]/+, *P* = 0.0003 by paired two-tailed *t*-test; Fig 3E).

## Discussion

Here, we present a case of anthropoid primate–specific placental gene expression induced by retroviral LTR insertion into the genome. Our study provides evidence that an LTR element found in a separate evolutionary lineage is capable of operating as a novel placental enhancer in another species. Placental tissue is globally hypomethylated and remarkably hospitable to endogenous retroviral elements [51, 52]. Endogenous retroviral LTRs have been co-opted for placenta-specific expression of hormones [35], endothelial factors [37, 53], and immune receptors [39]. Several of the THE1B-associated genes in our study that are expressed at significantly higher levels in human relative to mouse placenta have been previously implicated in human placental function, including the regulation of birth timing (*CGA* [54], *CRH* [1]) and adverse pregnancy outcomes like preeclampsia (*ADAM12*, *PGF* [55], *ZFAT* [56], *TUSC3* [57]) and recurrent miscarriage (*ADM* [58]). These findings suggest that THE1B elements likely influence the expression of a network of genes in human placenta and that this network of THE1B-associated genes may contribute to proper placental function.

Despite several lines of evidence implicating THE1B as an enhancer for *CRH*, we did not detect classical enhancer chromatin marks (H3K27Ac, H3K4me1) on this element in human term placental samples. It is possible that the THE1B near *CRH* enhances transcription using a different mechanism than the majority of vertebrate enhancers or, alternatively, that the positive enhancer signal is being diluted by the cells within the placenta that do not express *CRH*.

Previous studies of the species specificity of placental *CRH* expression have predominantly focused on the proximal promoter sequence of *CRH* [59, 60]. Scatena and Adler reported no expression of a luciferase reporter construct containing the 5-kb upstream region including THE1B in rat choriocarcinoma cells; this and other experiments led them to conclude that the species specificity of placental *CRH* expression was caused by *trans*-acting factors [59]. Our BAC transgenic mouse model incorporates both the human *CRH* and the mouse *Crh* promoter sequences and flanking regions; thus, differences in expression in this model are due to sequence differences rather than *trans*-acting factor availability. We observed differences in *CRH* expression when the THE1B sequence was intact (Tg[BAC1] and Tg[BAC2]), partially intact at the 5′ end (Tg[CR2]), and absent (Tg[CR1]), suggesting that the 5′ end of the THE1B LTR and its upstream insertion site are disproportionally active in the regulation of *CRH* in placenta.

The partially intact region of THE1B in Tg(CR2) mice contains an active binding site for the TF DLX3. DLX3 is present in mouse placenta and required for trophoblast differentiation; mice lacking *Dlx3* are unable to develop past embryonic day 10 due to placental failure [30]. The coexpression of *Dlx3* and *CRH* in Tg(BAC1) mouse placenta further associates DLX3 availability and THE1B-dependent expression of *CRH*. Notably, the DLX3 site occurs where the THE1B element joins the surrounding sequence, suggesting a possible mechanism for activation of this particular LTR upon retroviral insertion into the anthropoid primate common ancestral genome.

Our study is the first, to our knowledge, to provide evidence that placental expression of *CRH* in a nonprimate model alters gestation length. Surprisingly, human *CRH* expression in the placentas of our transgenic mice resulted in postterm rather than preterm birth. Although uterine prostaglandin F2α was significantly lower in Tg(BAC1) animals, progesterone withdrawal and expression of contractile-associated proteins were unaffected, indicating that CRH may alter birth timing without impeding luteolysis. Previous studies have shown that CRH plays a role in myometrial quiescence, inhibiting myometrial contractility at low CRH concentration [61–63]. This inhibition of uterine contractility is consistent with the postterm birth effect seen in our mouse model. These data suggest that CRH may be a factor contributing to the extended gestational period of anthropoid primates and that increased production of CRH in pregnancies ending prematurely may be an attempt to block the progression toward preterm labor and delivery.

The extraordinary diversity found in eutherian placental gene expression is thought to result from conflict between mother and fetus in determining optimal conditions for fetal development and maternal investment [64–66]. Placental tolerance to LTRs may play a role in this ongoing evolutionary conflict by providing a mechanism for rapid changes in gene expression. Our study demonstrates that retroviral insertion of an LTR such as THE1B can alter gene expression at the level of an individual gene and potentially across the entire placental tissue. Conservation of both THE1B and placental *CRH* expression in anthropoid primate species and the correlation of maternal serum CRH concentrations with gestation length support our assertion that the THE1B-CRH regulatory system is critical for birth timing.

## Materials and methods

### Human and rhesus tissue collection, RNA-seq, and transcriptome analysis

Human (GEO accession: GSE87726) and rhesus macaque (*Macaca mulatta*; GEO accession: GSE118284) placental transcriptomes were generated as previously described [67, 68]. Briefly, two human placentas were collected by cesarean section at 39 weeks 1 day and 39 weeks 2 days gestational age (IRB protocol: CCHMC IRB 2013–2243). Two macaque placentas were collected by cesarean section at 128 days and 131 days gestational age (80% completed gestation). RNA was extracted from placental biopsies with the TRIzol reagent (ThermoFisher Scientific) according to the manufacturer’s instructions. After passing initial quality control metrics, RNA-seq of the four samples was performed on an Illumina HiSeq machine using a paired-end approach with 50-bp reads, generating approximately 30 million paired reads per sample (human) and 15 million paired reads per sample (macaque). The reads were aligned and pseudocounted using kallisto [69]. To make the two transcriptomes comparable, we used the set of genes that are orthologous between the two species and recalculated the levels of expression of single genes in each species based only on this set. The levels are expressed on a TPM scale [70].

### PCR screening for THE1B-CRH fusion transcript in human and rhesus

Human and rhesus placental RNA was converted to cDNA using the QuantiTect Reverse Transcriptase Kit (Qiagen) according to manufacturer’s instructions. PCR screening was performed with primers specific to THE1B and *CRH* exon 2 (forward: 5′-CCTCCCCTACCATGTGAAAA-3′, reverse: 5′-GGAAGAAATCCAAGGGCT-3′) as well as with internal primers downstream of THE1B and in *CRH* exon 1 (forward: 5′- TGCTGTGCATAGCTTCTCCTC-3′, reverse: 5′-TGCCTCTGCTCCTGCATAAA-3′). PCR products were examined by agarose gel electrophoresis and confirmed by Sanger sequencing.

### Relative coexpression analysis of THE1B-associated genes

The statistical test for the effect of the presence of THE1B in the vicinity of the gene on the tissue-specific expression is described in detail by Pavlicev and colleagues [33]. Briefly, we compared the correlation between tissues based on the expression (expressed as the square root of the TPM value) of THE1B-associated genes to the correlation between tissues based on THE1B-absent genes. We repeated this procedure for all pair-wise tissue combinations. If the THE1B elements confer no particular status to the genes, this ratio corr_THE1B+_/corr_THE1B−_ should be close to 1 for every pair-wise tissue comparison. The significance of the effect was assessed based on the null distribution of the statistics for each pair of tissues compared, generated by randomly resampling 5,000 times the set of genes size matched to the number of THE1B-associated genes and calculating the statistics.

### THE1B capture array and analysis

Total RNA was purified from term placentas using a combination of TRIzol and Qiagen columns. RNA-seq libraries were prepared using the SureSelect Strand-Specific RNA Library Prep for Illumina Multiplexed Sequencing kit (Agilent). For capture sequencing, preamplified RNA-seq libraries were enriched for THE1B containing transcripts using a custom panel of 120 nt THE1B RNA oligos and the SureSelect^XT^ RNA Target Enrichment kit (Agilent). Libraries were sequenced as 75-bp paired-end reads on an Illumina HiSeq machine. Reads were aligned to the human genome (hg19) using Tophat2 (-I 100000—max-segment-intron 100000 -r 300) [71]. Data are available at GEO accession GSE118289.

### Annotation of human placenta–enriched genes

We compiled a collection of RNA-seq data for different human and mouse tissues from different resources, including (1) newly generated (GEO accession: GSE118289) and public (GEO accession: GSE43520) data for human placenta, (2) data for mouse placenta (GEO accession: GSE43520), and (3) data for different human tissues (brain, heart, kidney, liver, lung, ovary, skeletal muscle, and testis) from BodyMap 2.0 (GEO accession: GSE30611). Reference genome and gene annotation files for human (GRCh38) and mouse (GRCm38) were downloaded from the ENSEMBL database. Human placenta–enriched genes were identified by a procedure comparing human placenta against other human tissues and against mouse placenta. In brief, we first quantified gene expression levels for different samples as TPM using RSEM [72]. Then, we focused the analysis on the 13,068 1-to-1 orthologous genes between human and mouse according to ENSEMBL orthologue annotation. After the TPM values were normalized among different samples using the trimmed mean of M-values (TMM) method [73], differential expression analyses between human placenta and other analyzed human tissues and mouse placenta were performed using Rank Product method [74], with an FDR cutoff of 0.15. Finally, human placenta–enriched genes were annotated as genes that show significantly higher expression in human placenta against other human tissues and mouse placenta.

### Association analysis between human placenta–enriched genes and THE1B subfamily

We obtained repeat annotations for human THE1B subfamily from the RepeatMasker website (http://www.repeatmasker.org/) on May 27, 2016. To determine if the THE1B subfamily is associated with human placenta–enriched genes, we first determined the overlapping between THE1B repeat elements and promoter (defined as 20 kb upstream the longest transcript for each gene) for each protein-coding gene using the windowBed function of bedtools [75]. Then, the occurrences of THE1B in all these genes and in human placenta–enriched genes were counted. Finally, Binomial Test was performed to test if the THE1B subfamily is significantly associated with human placenta–enriched genes.

### Ethics statement

The Cincinnati Children’s Hospital Medical Center Institutional Animal Care and Use Committee approved all animal experiments for this study under IACUC protocol number 2017–0051. Animal procedures were designed and executed according to the guidelines of the National Institutes of Health.

### Establishment of transgenic mouse lines

Human BAC RP11-366K18 was obtained from the CHORI BACPAC repository. DNA was amplified and purified from bacteria using NucleoBond BAC kit (Clontech) and dissolved in polyamine buffer for microinjection. To generate lines Tg(BAC1) and Tg(BAC2), FVB/N zygotes were used for pronuclear injection of purified BAC DNA at 0.5 ng/ul and transferred into pseudopregnant CD-1 mice. The resulting pups were genotyped for presence of the human BAC with primers specific to human *CRH* (forward: 5′-TTTCTAATGTGAAAACTGCGTGAT-3′, reverse: 5′-ACACGTGGGAATTATGGGGG-3′). Transgenic lines were maintained as hemizygotes by crossing to C57BL/6 mice for 3–4 generations.

To delete the THE1B in the BAC transgenic mice, we selected 6 guide RNAs targeting the THE1B flanking region (3 per end), based on the web design tool (http://www.genome-engineering.org). Selected guide RNAs were cloned into pX458 vector (addgene #48138) and validated in HEK293 cells by T7E1 assay as described by Ran and colleagues [76]. Two validated guide RNAs (target sites: 5′-ATACATTTTGGATAATGATA-3′ and 5′-AGACAAATACAGATAAATTG-3′), along with *Cas9* mRNA, were introduced by cytoplasmic injection into zygotes obtained from Tg(BAC1) mice [77]. Injected embryos were transferred into pseudopregnant CD-1 mice. The resulting pups were screened for deletions to the THE1B region with primers flanking THE1B (forward: 5′-CAGTTTGTGTGCCTCTGCTG-3′, reverse: 5′-CTCCCATTGGGCTATCTGGG-3′) by PCR and Sanger sequencing.

### Mouse tissue collection and RNA isolation

Male mice were housed with females overnight to determine time of conception. Pregnant female mice were killed on the designated day of gestation, and placentas were harvested, dissected into labyrinth and junctional zone where indicated, and flash frozen in liquid nitrogen. Tissue from the tail of corresponding embryos was used to determine placental genotype with primers specific to human *CRH*, as described earlier. Hypothalamus, kidney, heart, liver, lung, and leg quadriceps muscle tissue were harvested from adult transgenic animals and littermate controls of both sexes, and tissues were immediately frozen in liquid nitrogen. Tissues were homogenized using stainless steel beads in a TissueLyser II apparatus (Qiagen), and RNA was purified using the RNeasy Mini Kit (Qiagen). Heart and skeletal muscle RNA was purified using the RNeasy Fibrous Tissue Mini Kit (Qiagen). Labyrinth and junctional zone RNA was purified using the TRIzol reagent (ThermoFisher Scientific) according to manufacturer’s instructions.

### qPCR for human *CRH* expression in transgenic mice

Mouse tissue RNA was converted to cDNA using the QuantiTect Reverse Transcriptase Kit (Qiagen) according to manufacturer’s instructions. Human *CRH* was quantified using the TaqMan system with TaqMan Gene Expression Master Mix (ThermoFisher Scientific) and specific probes for human *CRH* (Hs01921237_s1, ThermoFisher Scientific) and for mouse *Gapdh* as endogenous control (Mm99999915_g1, ThermoFisher Scientific). Labyrinth and junctional zone expression was quantified with specific probes for human *CRH* (Hs00174941_m1, ThermoFisher Scientific), mouse *Dlx3* (Mm00438428_m1, ThermoFisher Scientific), and eukaryotic 18S as endogenous control (catalog 4310893E, ThermoFisher Scientific). A 50-ng cDNA template was used per well, and samples were run in triplicate. qPCR reactions were run on an Applied Biosystems StepOnePlus Real-Time PCR instrument.

### Localization of human CRH by immunohistochemistry/immunofluorescence

Placentas of E18.5 pups were fixed in 4% paraformaldehyde in PBS overnight at 4 °C, washed in PBS, and dehydrated in 70% ethanol before embedding in paraffin. Paraffin sections were cut using a microtome. Sections were deparaffinized, incubated in citrate buffer pH 6.0 for antigen retrieval, and incubated in methanol with hydrogen peroxide for peroxidase activity removal. Following washes, slides were incubated with 5% goat serum and 4% fish skin gelatin for 60 minutes. Slides were incubated with CRH primary antibody (rabbit, Peninsula Lab T-4036, 1:1,000) overnight at 4 °C. Following washes, slides were incubated with secondary antibody (biotinylated goat anti-rabbit, Vector Lab BA-1000, 1:250) for 60 minutes. Tyramide amplification was performed with TSA cyanine 5 reagent kit (Perkin Elmer) according to manufacturer protocol.

### Quantification of gestation length in transgenic mice

Experimental animals and their respective control animals were generated by crossing hemizygous transgenic mice and subsequently crossing their homozygous offspring such that all study animals were the offspring of littermates. Male mice were housed with nulliparous female mice from approximately 1900 to 2200 hours once per week to precisely control time of conception. Pregnant females were monitored by observation from E17.5 until delivery. Pups were counted and weighed individually on the day of birth. Females were excluded from the study if there were fewer than four pups in the litter, if the female was older than 240 days of age on the date of conception, if the female was in labor for longer than 12 hours, or if the entire litter could not be delivered because of dystocia.

### Binding site prediction of placental TFs

Human and rhesus macaque placental transcriptome data were analyzed as previously stated. TFs expressed at 10 or greater TPM were populated into the predictive program CisBP [42]. Binding sites were predicted using the position-weight matrix (PWM)–LogOdds setting (threshold 8).

### Electrophoretic mobility shift assay for binding at THE1B

Immortalized extravillous trophoblast cell line HTR8/SVneo (obtained from the American Type Culture Collection, item number CRL-3271) was transfected with Lipofectamine3000 (ThermoFisher Scientific) and overexpression vector (DLX3 or GATA2 Myc-DDK vectors, Origene) for 72 hours according to manufacturer protocol. Cytoplasmic protein fraction was isolated by suspending cell pellet in cytoplasmic lysis buffer (10 mM HEPES, 10 mM KCl, 1.1 mM EDTA in dH_2_O) and adding 10% Nonidet-P40. Nuclear protein fraction was subsequently isolated by suspending nuclear pellet in nuclear lysis buffer (20 mM HEPES, 0.4 M KCl, 1 mM EDTA in dH_2_O). DNA probes were generated with IRDye700 at the 5′ end (GATA2 TCR probe: 5′-CACTTGATAACAGAAAGTGATAACTCT-3′ [78], DLX3 JRE probe: 5′-GGGGGGTAATTACAGGCCC-3′ [79], THE1B 5′ probe: 5′-GGATAATGATATGGTTAGA-3′). Binding reactions were performed using the Odyssey EMSA Buffer Kit (LICOR Biosciences) according to manufacturer protocol with primary antibody (monoclonal anti-FLAG M2, Sigma-Aldrich F3165) or isotype control (mouse IgG, Abcam ab37355) and run on 6% TBE gels with 0.5× TBE buffer (ThermoFisher Scientific). Gels were imaged on the Odyssey CLx Imaging System (LICOR Biosciences).

### ChIP and qPCR

Human placental tissue was homogenized in aliquots of 100 mg tissue per mL PBS with protease inhibitor (Abcam ab65621). Homogenate was transferred to a new tube with 18-gauge needle and syringe to ensure single cell suspension. Crosslinking was performed by adding 1.5% formaldehyde to suspension and incubating at room temperature for 10 minutes. Glycine was added to a final concentration of 125 mM to stop crosslinking reaction. Cells were washed twice with PBS and resuspended in SDS Lysis Buffer (Millipore). Cell lysate was sonicated to an average of 500-bp fragments with a Covaris S220 instrument at 175 peak power for 7 minutes. Immunoprecipitation was performed with the EZ-ChIP kit (Millipore) according to manufacturer protocol. Mouse anti-RNA pol II (Millipore 05-623B) and Control Mouse IgG antibodies (Millipore 12-371B) were used as a positive and negative control according to EZ-ChIP protocol. For DLX3 ChIP, rabbit anti-DLX3 (Abcam ab66390) and rabbit IgG isotype control (Abcam ab171870) were used at 5 μg per immunoprecipitation. qPCR reactions were run on an Applied Biosystems StepOnePlus Real-Time PCR instrument with primers specific to GAPDH (forward: 5′-TACTAGCGGTTTTACGGGCG-3′, reverse: 5′- TCGAACAGGAGGAGCAGAGAGCGA-3′), JRE (forward: 5′- TGACCTAAGGGTTGAAACAAGATAAG-3′, reverse: 5′- GGAAATTCCATCCAATGATTGA-3′) [45], distal region of JRE (forward: 5′- AGTTTCTTTGTGGATGAAGAGATAGACG-3′, reverse: 5′- TTTTCCGAACTTCAAAGGCCCTG-3′) [45], and the 5′ end of the THE1B near CRH (forward: 5′- TTTCTAATGTGAAAACTGCGTGA-3′, reverse: 5′- ACACGTGGGAATTATGGGGG-3′).

### Statistical analysis

Statistical analysis was performed with GraphPad Prism (GraphPad Software) and R Statistical Programming Language. Specific statistical tests are described above.

## Supporting information

S1 Data Numerical data and statistical analysis for Figs 1D, 2B, 2C, 2D, 2G, 2H, 2I, 3C and 3E and for S2A, S2B, S2C, S3A, S3B and S5 in separate sheets. (XLSX)

S1 Text Materials and methods for supporting information. (DOCX)

S1 Fig THE1B near *CRH* is not associated with classical enhancer chromatin marks. (A) Comparison of reads from human placental transcriptome pre-THE1B capture (RNA-seq) and post-THE1B capture (Capture-seq) to ChIP-seq reads for H3K27ac and H3K4me1 at the THE1B near *CRH*. This THE1B element lacks these chromatin marks, which are commonly associated with enhancers. (B) Comparison of reads from human placental transcriptome pre-THE1B capture (RNA-seq) and post-THE1B capture (Capture-seq) to ChIP-seq reads for H3K27ac and H3K4me1 at several THE1B elements near the *GRIK1* gene. Enrichment of these chromatin marks may indicate enhancer activity. Raw data for A and B can be found at GEO accession number GSE118289. ChIP-seq, chromatin immunoprecipitation sequencing; GEO, Gene Expression Omnibus; H3K4me1, histone H3 lysine 4 monomethylation; H3K27ac, histone H3 lysine 27 acetylation; RNA-seq, RNA sequencing; THE1B, transposon-like human element 1B.
(TIF)

S2 Fig Postterm birth phenotype is independent of progesterone and contractile-associated protein changes but may involve prostaglandin F2α. (A) Progesterone concentration in maternal serum at E18.5. (*n* = 5–10 per group, *P* = 0.6342 by one-way ANOVA. Error bars indicate the standard error of the mean.) (B) Prostaglandin F2α concentration in uterine tissue at E18.5 is significantly lower in Tg(BAC1) litters and approaches significance in Tg(BAC2) litters. (*n* = 7–11 per group, *P* = 0.0021 by one-way ANOVA. When compared to +/+, *P* = 0.0012 for Tg[BAC1]/Tg[BAC1] and *P* = 0.0635 for Tg[BAC2]/Tg[BAC2] by post hoc Dunnett’s multiple comparisons test. Error bars indicate the standard error of the mean.) (C) Expression of contractile-associated proteins in uterus of Tg(BAC1) and control animals measured by RNA-seq at E18.5. (*n* = 4 +/+, *n* = 3 Tg[BAC1]/Tg[BAC1]. All error bars indicate the standard error of the mean and were analyzed by unpaired two-tailed *t*-test. *Oxtr*, *P* = 0.6795. *Gja1*, *P* = 0.3005. *Cav1*, *P* = 0.4035. *Ptgs1*, *P* = 0.9313.) *Ptgs2* expression was uniformly very low (TPM < 3.5 for all samples, *P* = 0.6485). Raw data can be found at GEO accession GSE118283. Numerical data for A, B, and C can be found in S1 Data. E18.5, embryonic day 18.5; GEO, Gene Expression Omnibus; RNA-seq, RNA sequencing; TPM, transcripts per million.
(TIF)

S3 Fig Hypothalamic-pituitary-adrenal axis function in transgenic mice is comparable to control. (A) Relative expression of mouse *Crh* in hypothalamus. Expression of *Crh* is down-regulated to compensate for addition of *CRH* expression in transgenic mouse hypothalamus. (*n* = 3 all groups, *P* = 0.0068 by one-way ANOVA. When compared to +/+, *P* = 0.0340 for Tg[BAC1]/+ and *P* = 0.0030 for Tg[CR2]/+ by post hoc Dunnett’s multiple comparisons test. Error bars indicate the standard error of the mean.) (B) Nadir and peak corticosterone (“CORT”) concentration in the serum of male (left) and female (right) transgenic mice. (*n* ≥ 3 all groups, *P* = 0.6568 for males and *P* = 0.9978 for females by two-way ANOVA, with genotype as the source of variation. Error bars indicate the standard error of the mean.) Numerical data for A and B can be found in S1 Data.
(TIF)

S4 Fig *TRIM55* expression is altered by deletion of THE1B from the human BAC. Gene expression from the human BAC in mouse placenta measured by paired-end RNA-seq. The only other protein-coding gene on the BAC, *TRIM55*, is located in a tail-to-tail orientation very close to *CRH*. Tg(BAC1) animals express *TRIM55* and *CRH* in placenta, whereas Tg(CR1) animals do not. Red bar, highlighted region of BAC RP11-366K18 shown to scale. Raw data can be found at GEO accession GSE118283. BAC, bacterial artificial chromosome; GEO, Gene Expression Omnibus; RNA-seq, RNA sequencing; THE1B, transposon-like human element 1B.
(TIF)

S5 Fig Gestation length of litters hemizygous for Tg(CR2) is not significantly different from wild-type control. Gestation length of Tg(CR2)/+ and control litters. Although Tg(CR2) transgenic animals retain a small amount of placental *CRH* expression, litters receiving Tg(CR2) only from the father have no difference in gestation length when compared to wild-type control litters. (*n* = 14 +/+, *n* = 12 Tg[CR2]/+, *P* = 0.6397 by unpaired two-tailed *t*-test. Error bars indicate standard deviation.) Numerical data can be found in S1 Data.
(TIF)

S1 Table THE1B-CRH novel splice site is conserved in anthropoid primate species. Red text denotes predicted splice sites for anthropoid primate species. Primate genome sequences from UCSC Genome Browser, accessed February 27, 2018. CRH, corticotropin-releasing hormone; THE1B, transposon-like human element 1B.
(DOCX)

## Abbreviations

BAC: bacterial artificial chromosome
*Cav1*: caveolin-1
ChIP-seq: chromatin immunoprecipitation sequencing
COX-1: cyclooxygenase 1
COX-2: cyclooxygenase 2
CRH: corticotropin-releasing hormone
CRISPR/Cas9: clustered regularly interspaced short palindromic repeat/CRISPR-associated 9
DLX3: distal-less homeobox 3
E18.5: embryonic day 18.5
Endom. strom.: endometrial stromal cells
H3K27ac: histone H3 lysine 27 acetylation
H3K4me1: histone H3 lysine 4 monomethylation
JRE: human glycoprotein hormone α-subunit junctional regulatory element
LTR: long terminal repeat
ns: not significant
*Oxtr*: oxytocin receptor
PAM: protospacer adjacent motif
PWM: position-weight matrix
qPCR: quantitative PCR
RBC: red blood cell
RNA-seq: RNA sequencing
TF: transcription factor
THE1B: transposon-like human element 1B
TMM: trimmed mean of M-values
TPM: transcripts per million

## References

[1] McLean M, Bisits A, Davies J, Woods R, Lowry P, Smith R. A placental clock controlling the length of human pregnancy. Nat Med. 1995;1(5):460–3. Epub 1995/05/01. . 7585095 10.1038/nm0595-460

[2] Wadhwa PD, Porto M, Garite TJ, Chicz-DeMet A, Sandman CA. Maternal corticotropin-releasing hormone levels in the early third trimester predict length of gestation in human pregnancy. Am J Obstet Gynecol. 1998;179(4):1079–85. Epub 1998/10/28. . 9790402 10.1016/s0002-9378(98)70219-4

[3] Wadhwa PD, Garite TJ, Porto M, Glynn L, Chicz-DeMet A, Dunkel-Schetter C, et al Placental corticotropin-releasing hormone (CRH), spontaneous preterm birth, and fetal growth restriction: a prospective investigation. Am J Obstet Gynecol. 2004;191(4):1063–9. Epub 2004/10/28. 10.1016/j.ajog.2004.06.070 . 15507922

[4] Sandman CA, Glynn L, Schetter CD, Wadhwa P, Garite T, Chicz-DeMet A, et al Elevated maternal cortisol early in pregnancy predicts third trimester levels of placental corticotropin releasing hormone (CRH): priming the placental clock. Peptides. 2006;27(6):1457–63. Epub 2005/11/29. 10.1016/j.peptides.2005.10.002 . 16309788

[5] Schulkin J. Evolutionary conservation of glucocorticoids and corticotropin releasing hormone: behavioral and physiological adaptations. Brain Res. 2011;1392:27–46. Epub 2011/03/31. 10.1016/j.brainres.2011.03.055 . 21447324

[6] Robinson BG, Arbiser JL, Emanuel RL, Majzoub JA. Species-specific placental corticotropin releasing hormone messenger RNA and peptide expression. Mol Cell Endocrinol. 1989;62(2):337–41. Epub 1989/04/01. . 2787253 10.1016/0303-7207(89)90022-1

[7] Goland RS, Wardlaw SL, Fortman JD, Stark RI. Plasma corticotropin-releasing factor concentrations in the baboon during pregnancy. Endocrinology. 1992;131(4):1782–6. Epub 1992/10/01. 10.1210/endo.131.4.1396323 . 1396323

[8] Smith R, Chan EC, Bowman ME, Harewood WJ, Phippard AF. Corticotropin-releasing hormone in baboon pregnancy. J Clin Endocrinol Metab. 1993;76(4):1063–8. Epub 1993/04/01. 10.1210/jcem.76.4.8473382 . 8473382

[9] Giussani DA, Winter JA, Jenkins SL, Tame JD, Abrams LM, Ding XY, et al Changes in fetal plasma corticotropin-releasing hormone during androstenedione-induced labor in the rhesus monkey: lack of an effect on the fetal hypothalamo-pituitary-adrenal axis. Endocrinology. 1998;139(6):2803–10. Epub 1998/06/02. 10.1210/endo.139.6.6044 . 9607787

[10] Smith R, Wickings EJ, Bowman ME, Belleoud A, Dubreuil G, Davies JJ, et al Corticotropin-releasing hormone in chimpanzee and gorilla pregnancies. J Clin Endocrinol Metab. 1999;84(8):2820–5. Epub 1999/08/12. 10.1210/jcem.84.8.5906 . 10443686

[11] Bowman ME, Lopata A, Jaffe RB, Golos TG, Wickings J, Smith R. Corticotropin-releasing hormone-binding protein in primates. Am J Primatol. 2001;53(3):123–30. Epub 2001/03/20. 10.1002/1098-2345(200103)53:3<123::AID-AJP3>3.0.CO;2-V . 11253847

[12] Power ML, Bowman ME, Smith R, Ziegler TE, Layne DG, Schulkin J, et al Pattern of maternal serum corticotropin-releasing hormone concentration during pregnancy in the common marmoset (Callithrix jacchus). Am J Primatol. 2006;68(2):181–8. Epub 2006/01/24. 10.1002/ajp.20215 . 16429419

[13] Power ML, Schulkin J. Functions of corticotropin-releasing hormone in anthropoid primates: from brain to placenta. Am J Hum Biol. 2006;18(4):431–47. Epub 2006/06/22. 10.1002/ajhb.20521 . 16788901

[14] Power ML, Williams LE, Gibson SV, Schulkin J, Helfers J, Zorrilla EP. Pattern of maternal circulating CRH in laboratory-housed squirrel and owl monkeys. Am J Primatol. 2010;72(11):1004–12. Epub 2010/09/28. 10.1002/ajp.20850 . 20872786 PMC2947327

[15] Brunet TD, Doolittle WF. Multilevel Selection Theory and the Evolutionary Functions of Transposable Elements. Genome Biol Evol. 2015;7(8):2445–57. Epub 2015/08/09. 10.1093/gbe/evv152 . 26253318 PMC4558868

[16] Chuong EB, Elde NC, Feschotte C. Regulatory activities of transposable elements: from conflicts to benefits. Nat Rev Genet. 2017;18(2):71–86. Epub 2016/11/22. 10.1038/nrg.2016.139 . 27867194 PMC5498291

[17] Mikkelsen TS, Wakefield MJ, Aken B, Amemiya CT, Chang JL, Duke S, et al Genome of the marsupial Monodelphis domestica reveals innovation in non-coding sequences. Nature. 2007;447(7141):167–77. Epub 2007/05/15. 10.1038/nature05805 . 17495919

[18] Wang T, Zeng J, Lowe CB, Sellers RG, Salama SR, Yang M, et al Species-specific endogenous retroviruses shape the transcriptional network of the human tumor suppressor protein p53. Proc Natl Acad Sci U S A. 2007;104(47):18613–8. Epub 2007/11/16. 10.1073/pnas.0703637104 . 18003932 PMC2141825

[19] Lowe CB, Haussler D. 29 mammalian genomes reveal novel exaptations of mobile elements for likely regulatory functions in the human genome. PLoS ONE. 2012;7(8):e43128 10.1371/journal.pone.0043128 . 22952639 PMC3428314

[20] Jacques PE, Jeyakani J, Bourque G. The majority of primate-specific regulatory sequences are derived from transposable elements. PLoS Genet. 2013;9(5):e1003504 10.1371/journal.pgen.1003504 . 23675311 PMC3649963

[21] Sundaram V, Cheng Y, Ma Z, Li D, Xing X, Edge P, et al Widespread contribution of transposable elements to the innovation of gene regulatory networks. Genome Res. 2014;24(12):1963–76. Epub 2014/10/17. 10.1101/gr.168872.113 . 25319995 PMC4248313

[22] Chuong EB, Rumi MA, Soares MJ, Baker JC. Endogenous retroviruses function as species-specific enhancer elements in the placenta. Nat Genet. 2013;45(3):325–9. Epub 2013/02/12. 10.1038/ng.2553 . 23396136 PMC3789077

[23] Faulkner GJ, Kimura Y, Daub CO, Wani S, Plessy C, Irvine KM, et al The regulated retrotransposon transcriptome of mammalian cells. Nat Genet. 2009;41(5):563–71. Epub 2009/04/21. 10.1038/ng.368 . 19377475

[24] Emera D, Casola C, Lynch VJ, Wildman DE, Agnew D, Wagner GP. Convergent evolution of endometrial prolactin expression in primates, mice, and elephants through the independent recruitment of transposable elements. Mol Biol Evol. 2012;29(1):239–47. Epub 2011/08/05. 10.1093/molbev/msr189 . 21813467

[25] Macfarlan TS, Gifford WD, Driscoll S, Lettieri K, Rowe HM, Bonanomi D, et al Embryonic stem cell potency fluctuates with endogenous retrovirus activity. Nature. 2012;487(7405):57–63. Epub 2012/06/23. 10.1038/nature11244 . 22722858 PMC3395470

[26] Gifford WD, Pfaff SL, Macfarlan TS. Transposable elements as genetic regulatory substrates in early development. Trends Cell Biol. 2013;23(5):218–26. Epub 2013/02/16. 10.1016/j.tcb.2013.01.001 . 23411159 PMC4034679

[27] Xie M, Hong C, Zhang B, Lowdon RF, Xing X, Li D, et al DNA hypomethylation within specific transposable element families associates with tissue-specific enhancer landscape. Nat Genet. 2013;45(7):836–41. Epub 2013/05/28. 10.1038/ng.2649 . 23708189 PMC3695047

[28] Thompson PJ, Macfarlan TS, Lorincz MC. Long Terminal Repeats: From Parasitic Elements to Building Blocks of the Transcriptional Regulatory Repertoire. Mol Cell. 2016;62(5):766–76. Epub 2016/06/04. 10.1016/j.molcel.2016.03.029 . 27259207 PMC4910160

[29] Smit AF. Identification of a new, abundant superfamily of mammalian LTR-transposons. Nucleic Acids Res. 1993;21(8):1863–72. Epub 1993/04/25. . 8388099 10.1093/nar/21.8.1863 PMC309426

[30] Morasso MI, Grinberg A, Robinson G, Sargent TD, Mahon KA. Placental failure in mice lacking the homeobox gene Dlx3. Proc Natl Acad Sci U S A. 1999;96(1):162–7. Epub 1999/01/06. . 9874789 10.1073/pnas.96.1.162 PMC15110

[31] Lamprecht B, Walter K, Kreher S, Kumar R, Hummel M, Lenze D, et al Derepression of an endogenous long terminal repeat activates the CSF1R proto-oncogene in human lymphoma. Nat Med. 2010;16(5):571–9, 1p following 9. Epub 2010/05/04. 10.1038/nm.2129 . 20436485

[32] Young JM, Whiddon JL, Yao Z, Kasinathan B, Snider L, Geng LN, et al DUX4 binding to retroelements creates promoters that are active in FSHD muscle and testis. PLoS Genet. 2013;9(11):e1003947 10.1371/journal.pgen.1003947 . 24278031 PMC3836709

[33] Pavlicev M, Hiratsuka K, Swaggart KA, Dunn C, Muglia L. Detecting endogenous retrovirus-driven tissue-specific gene transcription. Genome Biol Evol. 2015;7(4):1082–97. Epub 2015/03/15. 10.1093/gbe/evv049 . 25767249 PMC4419796

[34] Schulte AM, Lai S, Kurtz A, Czubayko F, Riegel AT, Wellstein A. Human trophoblast and choriocarcinoma expression of the growth factor pleiotrophin attributable to germ-line insertion of an endogenous retrovirus. Proc Natl Acad Sci U S A. 1996;93(25):14759–64. Epub 1996/12/10. . 8962128 10.1073/pnas.93.25.14759 PMC26209

[35] Bieche I, Laurent A, Laurendeau I, Duret L, Giovangrandi Y, Frendo JL, et al Placenta-specific INSL4 expression is mediated by a human endogenous retrovirus element. Biol Reprod. 2003;68(4):1422–9. Epub 2003/02/28. 10.1095/biolreprod.102.010322 . 12606452

[36] Prudhomme S, Bonnaud B, Mallet F. Endogenous retroviruses and animal reproduction. Cytogenet Genome Res. 2005;110(1–4):353–64. Epub 2005/08/12. 10.1159/000084967 . 16093687

[37] Huh JW, Ha HS, Kim DS, Kim HS. Placenta-restricted expression of LTR-derived NOS3. Placenta. 2008;29(7):602–8. Epub 2008/05/14. 10.1016/j.placenta.2008.04.002 . 18474398

[38] Cohen CJ, Lock WM, Mager DL. Endogenous retroviral LTRs as promoters for human genes: a critical assessment. Gene. 2009;448(2):105–14. Epub 2009/07/07. 10.1016/j.gene.2009.06.020 . 19577618

[39] Cohen CJ, Rebollo R, Babovic S, Dai EL, Robinson WP, Mager DL. Placenta-specific expression of the interleukin-2 (IL-2) receptor beta subunit from an endogenous retroviral promoter. J Biol Chem. 2011;286(41):35543–52. Epub 2011/08/26. 10.1074/jbc.M111.227637 . 21865161 PMC3195601

[40] Centner T, Yano J, Kimura E, McElhinny AS, Pelin K, Witt CC, et al Identification of muscle specific ring finger proteins as potential regulators of the titin kinase domain. J Mol Biol. 2001;306(4):717–26. Epub 2001/03/13. 10.1006/jmbi.2001.4448 . 11243782

[41] Perera S, Holt MR, Mankoo BS, Gautel M. Developmental regulation of MURF ubiquitin ligases and autophagy proteins nbr1, p62/SQSTM1 and LC3 during cardiac myofibril assembly and turnover. Dev Biol. 2011;351(1):46–61. Epub 2010/12/28. 10.1016/j.ydbio.2010.12.024 . 21185285 PMC3047806

[42] Weirauch MT, Yang A, Albu M, Cote AG, Montenegro-Montero A, Drewe P, et al Determination and inference of eukaryotic transcription factor sequence specificity. Cell. 2014;158(6):1431–43. Epub 2014/09/13. 10.1016/j.cell.2014.08.009 . 25215497 PMC4163041

[43] Paul S, Home P, Bhattacharya B, Ray S. GATA factors: Master regulators of gene expression in trophoblast progenitors. Placenta. 2017;60 Suppl 1:S61–s6. Epub 2017/05/21. 10.1016/j.placenta.2017.05.005 . 28526138 PMC7021224

[44] Han L, Dias Figueiredo M, Berghorn KA, Iwata TN, Clark-Campbell PA, Welsh IC, et al Analysis of the gene regulatory program induced by the homeobox transcription factor distal-less 3 in mouse placenta. Endocrinology. 2007;148(3):1246–54. Epub 2006/11/18. 10.1210/en.2006-1356 . 17110422

[45] Li S, Roberson MS. Dlx3 and GCM-1 functionally coordinate the regulation of placental growth factor in human trophoblast-derived cells. J Cell Physiol. 2017;232(10):2900–14. Epub 2016/12/21. 10.1002/jcp.25752 . 27996093

[46] Jolma A, Yan J, Whitington T, Toivonen J, Nitta KR, Rastas P, et al DNA-binding specificities of human transcription factors. Cell. 2013;152(1–2):327–39. Epub 2013/01/22. 10.1016/j.cell.2012.12.009 . 23332764

[47] Berghorn KA, Clark PA, Encarnacion B, Deregis CJ, Folger JK, Morasso MI, et al Developmental expression of the homeobox protein Distal-less 3 and its relationship to progesterone production in mouse placenta. J Endocrinol. 2005;186(2):315–23. Epub 2005/08/05. 10.1677/joe.1.06217 . 16079257

[48] Riley SC, Walton JC, Herlick JM, Challis JR. The localization and distribution of corticotropin-releasing hormone in the human placenta and fetal membranes throughout gestation. J Clin Endocrinol Metab. 1991;72(5):1001–7. Epub 1991/05/01. 10.1210/jcem-72-5-1001 . 2022703

[49] Goland RS, Jozak S, Warren WB, Conwell IM, Stark RI, Tropper PJ. Elevated levels of umbilical cord plasma corticotropin-releasing hormone in growth-retarded fetuses. J Clin Endocrinol Metab. 1993;77(5):1174–9. Epub 1993/11/01. 10.1210/jcem.77.5.8077309 . 8077309

[50] Chui A, Pathirage NA, Johnson B, Cocquebert M, Fournier T, Evain-Brion D, et al Homeobox gene distal-less 3 is expressed in proliferating and differentiating cells of the human placenta. Placenta. 2010;31(8):691–7. Epub 2010/06/15. 10.1016/j.placenta.2010.05.003 . 20542333

[51] Schroeder DI, Blair JD, Lott P, Yu HO, Hong D, Crary F, et al The human placenta methylome. Proc Natl Acad Sci U S A. 2013;110(15):6037–42. Epub 2013/03/27. 10.1073/pnas.1215145110 . 23530188 PMC3625261

[52] Schroeder DI, Jayashankar K, Douglas KC, Thirkill TL, York D, Dickinson PJ, et al Early Developmental and Evolutionary Origins of Gene Body DNA Methylation Patterns in Mammalian Placentas. PLoS Genet. 2015;11(8):e1005442 10.1371/journal.pgen.1005442 . 26241857 PMC4524645

[53] Landry JR, Mager DL. Functional analysis of the endogenous retroviral promoter of the human endothelin B receptor gene. J Virol. 2003;77(13):7459–66. Epub 2003/06/14. 10.1128/JVI.77.13.7459-7466.2003 . 12805445 PMC164795

[54] Fournier T, Guibourdenche J, Evain-Brion D. Review: hCGs: different sources of production, different glycoforms and functions. Placenta. 2015;36 Suppl 1:S60–5. Epub 2015/02/25. 10.1016/j.placenta.2015.02.002 . 25707740

[55] Myatt L, Clifton RG, Roberts JM, Spong CY, Hauth JC, Varner MW, et al First-trimester prediction of preeclampsia in nulliparous women at low risk. Obstet Gynecol. 2012;119(6):1234–42. Epub 2012/05/24. 10.1097/AOG.0b013e3182571669 . 22617589 PMC3360523

[56] Barbaux S, Gascoin-Lachambre G, Buffat C, Monnier P, Mondon F, Tonanny MB, et al A genome-wide approach reveals novel imprinted genes expressed in the human placenta. Epigenetics. 2012;7(9):1079–90. Epub 2012/08/17. 10.4161/epi.21495 . 22894909 PMC3466192

[57] Yuen RK, Avila L, Penaherrera MS, von Dadelszen P, Lefebvre L, Kobor MS, et al Human placental-specific epipolymorphism and its association with adverse pregnancy outcomes. PLoS ONE. 2009;4(10):e7389 10.1371/journal.pone.0007389 . 19838307 PMC2760756

[58] Banerjee P, Ghosh S, Dutta M, Subramani E, Khalpada J, Roychoudhury S, et al Identification of key contributory factors responsible for vascular dysfunction in idiopathic recurrent spontaneous miscarriage. PLoS ONE. 2013;8(11):e80940 10.1371/journal.pone.0080940 . 24260517 PMC3829935

[59] Scatena CD, Adler S. Trans-acting factors dictate the species-specific placental expression of corticotropin-releasing factor genes in choriocarcinoma cell lines. Endocrinology. 1996;137(7):3000–8. Epub 1996/07/01. 10.1210/endo.137.7.8770924 . 8770924

[60] Scatena CD, Adler S. Characterization of a human-specific regulator of placental corticotropin-releasing hormone. Mol Endocrinol. 1998;12(8):1228–40. Epub 1998/08/26. 10.1210/mend.12.8.0150 . 9717848

[61] Aggelidou E, Hillhouse EW, Grammatopoulos DK. Up-regulation of nitric oxide synthase and modulation of the guanylate cyclase activity by corticotropin-releasing hormone but not urocortin II or urocortin III in cultured human pregnant myometrial cells. Proc Natl Acad Sci U S A. 2002;99(5):3300–5. Epub 2002/02/21. 10.1073/pnas.052296399 . 11854458 PMC122513

[62] Tyson EK, Smith R, Read M. Evidence that corticotropin-releasing hormone modulates myometrial contractility during human pregnancy. Endocrinology. 2009;150(12):5617–25. Epub 2009/10/23. 10.1210/en.2009-0348 . 19846610

[63] Kim JY, Wu WH, Jun JH, Sohn J, Seo YS. Effects of corticotropin-releasing hormone on the expression of adenosine triphosphate-sensitive potassium channels (Kir6.1/SUR2B) in human term pregnant myometrium. Obstet Gynecol Sci. 2018;61(1):14–22. Epub 2018/01/27. 10.5468/ogs.2018.61.1.14 . 29372145 PMC5780309

[64] Haig D. Genetic conflicts in human pregnancy. Q Rev Biol. 1993;68(4):495–532. Epub 1993/12/01. . 8115596 10.1086/418300

[65] Chuong EB, Hannibal RL, Green SL, Baker JC. Evolutionary perspectives into placental biology and disease. Appl Transl Genom. 2013;2:64–9. Epub 2013/09/18. 10.1016/j.atg.2013.07.001 . 27896057 PMC5121266

[66] Roberts RM, Green JA, Schulz LC. The evolution of the placenta. Reproduction. 2016;152(5):R179–89. Epub 2016/08/04. 10.1530/REP-16-0325 . 27486265 PMC5033709

[67] Eidem HR, Rinker DC, Ackerman WEt, Buhimschi IA, Buhimschi CS, Dunn-Fletcher C, et al Comparing human and macaque placental transcriptomes to disentangle preterm birth pathology from gestational age effects. Placenta. 2016;41:74–82. Epub 2016/05/22. 10.1016/j.placenta.2016.03.006 . 27208410 PMC11580153

[68] Pavlicev M, Wagner GP, Chavan AR, Owens K, Maziarz J, Dunn-Fletcher C, et al Single-cell transcriptomics of the human placenta: inferring the cell communication network of the maternal-fetal interface. Genome Res. 2017;27(3):349–61. Epub 2017/02/09. 10.1101/gr.207597.116 . 28174237 PMC5340963

[69] Bray NL, Pimentel H, Melsted P, Pachter L. Near-optimal probabilistic RNA-seq quantification. Nat Biotechnol. 2016;34(5):525–7. Epub 2016/04/05. 10.1038/nbt.3519 . 27043002

[70] Wagner GP, Kin K, Lynch VJ. Measurement of mRNA abundance using RNA-seq data: RPKM measure is inconsistent among samples. Theory Biosci. 2012;131(4):281–5. Epub 2012/08/09. 10.1007/s12064-012-0162-3 . 22872506

[71] Kim D, Pertea G, Trapnell C, Pimentel H, Kelley R, Salzberg SL. TopHat2: accurate alignment of transcriptomes in the presence of insertions, deletions and gene fusions. Genome Biol. 2013;14(4):R36 Epub 2013/04/27. 10.1186/gb-2013-14-4-r36 . 23618408 PMC4053844

[72] Li B, Dewey CN. RSEM: accurate transcript quantification from RNA-Seq data with or without a reference genome. BMC Bioinformatics. 2011;12:323 Epub 2011/08/06. 10.1186/1471-2105-12-323 . 21816040 PMC3163565

[73] Robinson MD, McCarthy DJ, Smyth GK. edgeR: a Bioconductor package for differential expression analysis of digital gene expression data. Bioinformatics. 2010;26(1):139–40. Epub 2009/11/17. 10.1093/bioinformatics/btp616 . 19910308 PMC2796818

[74] Hong F, Breitling R, McEntee CW, Wittner BS, Nemhauser JL, Chory J. RankProd: a bioconductor package for detecting differentially expressed genes in meta-analysis. Bioinformatics. 2006;22(22):2825–7. Epub 2006/09/20. 10.1093/bioinformatics/btl476 . 16982708

[75] Quinlan AR, Hall IM. BEDTools: a flexible suite of utilities for comparing genomic features. Bioinformatics. 2010;26(6):841–2. Epub 2010/01/30. 10.1093/bioinformatics/btq033 . 20110278 PMC2832824

[76] Ran FA, Hsu PD, Wright J, Agarwala V, Scott DA, Zhang F. Genome engineering using the CRISPR-Cas9 system. Nat Protoc. 2013;8(11):2281–308. Epub 2013/10/26. 10.1038/nprot.2013.143 . 24157548 PMC3969860

[77] Yuan CL, Hu YC. A Transgenic Core Facility's Experience in Genome Editing Revolution. Adv Exp Med Biol. 2017;1016:75–90. Epub 2017/11/14. 10.1007/978-3-319-63904-8_4 . 29130154

[78] Hahn CN, Chong CE, Carmichael CL, Wilkins EJ, Brautigan PJ, Li XC, et al Heritable GATA2 mutations associated with familial myelodysplastic syndrome and acute myeloid leukemia. Nat Genet. 2011;43(10):1012–7. Epub 2011/09/06. 10.1038/ng.913 . 21892162 PMC3184204

[79] Duverger O, Lee D, Hassan MQ, Chen SX, Jaisser F, Lian JB, et al Molecular consequences of a frameshifted DLX3 mutant leading to Tricho-Dento-Osseous syndrome. J Biol Chem. 2008;283(29):20198–208. Epub 2008/05/22. 10.1074/jbc.M709562200 . 18492670 PMC2459267

