# Supplementary material for: Anthropoid primate–specific retroviral element THE1B controls expression of CRH in placenta and alters gestation length
Source: PLoS Biol. 2018 Sep 19;16(9):e2006337. doi: 10.1371/journal.pbio.2006337 (PMC6166974; doi:10.1371/journal.pbio.2006337)
Supplement: S1 Text — (DOCX) [file pbio.2006337.s002.docx]

**SI MATERIALS AND METHODS**

**ChIP-seq for histone modifications in human term placenta**

Human term placental samples were acquired and processed as described in Materials and Methods. Chromatin immunoprecipitation was performed using the MAGnify ChIP Kit (ThermoFisher) and antibodies to H3K4me1 (ab8895, Abcam) and H3K27ac (ab4729, Abcam). ChIP-seq data are available at GEO accession number GSE118289.

**Measurement of serum progesterone**

Mice were anesthetized with isofluorane on embryonic day 18.5 (E18.5) and cardiac puncture was performed to obtain maternal blood. Blood was centrifuged to isolate serum. ELISA was performed using the Progesterone Mouse/Rat ELISA Kit (BioVendor).

**Measurement of uterine prostaglandin F2α**

Uterine tissue was harvested at E18.5 and weighed to provide an initial tissue weight. Prostaglandins were extracted from tissue by homogenization in 100% ethanol, centrifugation and transfer of liquid suspension, and evaporation of ethanol under inert gas. ELISA was performed using the PGF2-alpha EIA Kit (Oxford Biomedical Research) and results were normalized to initial tissue weight.

**Measurement of serum corticosterone**

Submandibular bleeds were performed in mice at nadir (8:00 AM) and peak (7:00 PM), with 7 days in between bleeds. The blood was centrifuged at 15 000 RPM for 6 minutes and the plasma was removed and stored at -20*C until ELISA was performed. ELISA was performed using the Corticosterone Mouse/Rat ELISA Kit (BioVendor).

**Uterus and placenta RNA-sequencing and analysis**

Uterine and placental tissue were harvested at E18.5 and RNA was extracted with the TRIzol reagent (ThermoFisher Scientific) according to manufacturer’s instructions. After passing initial quality control metrics, RNA-seq was performed on an Illumina HiSeq machine using a paired-end approach with 75 bp reads, generating approximately 30 million paired reads per sample. Reads were aligned as described in Materials and Methods. Data are available at GEO accession GSE118283.
