# Supplementary figures and images for: Anthropoid primate–specific retroviral element THE1B controls expression of CRH in placenta and alters gestation length
Source: PLoS Biol. 2018 Sep 19;16(9):e2006337. doi: 10.1371/journal.pbio.2006337 (PMC6166974; doi:10.1371/journal.pbio.2006337)

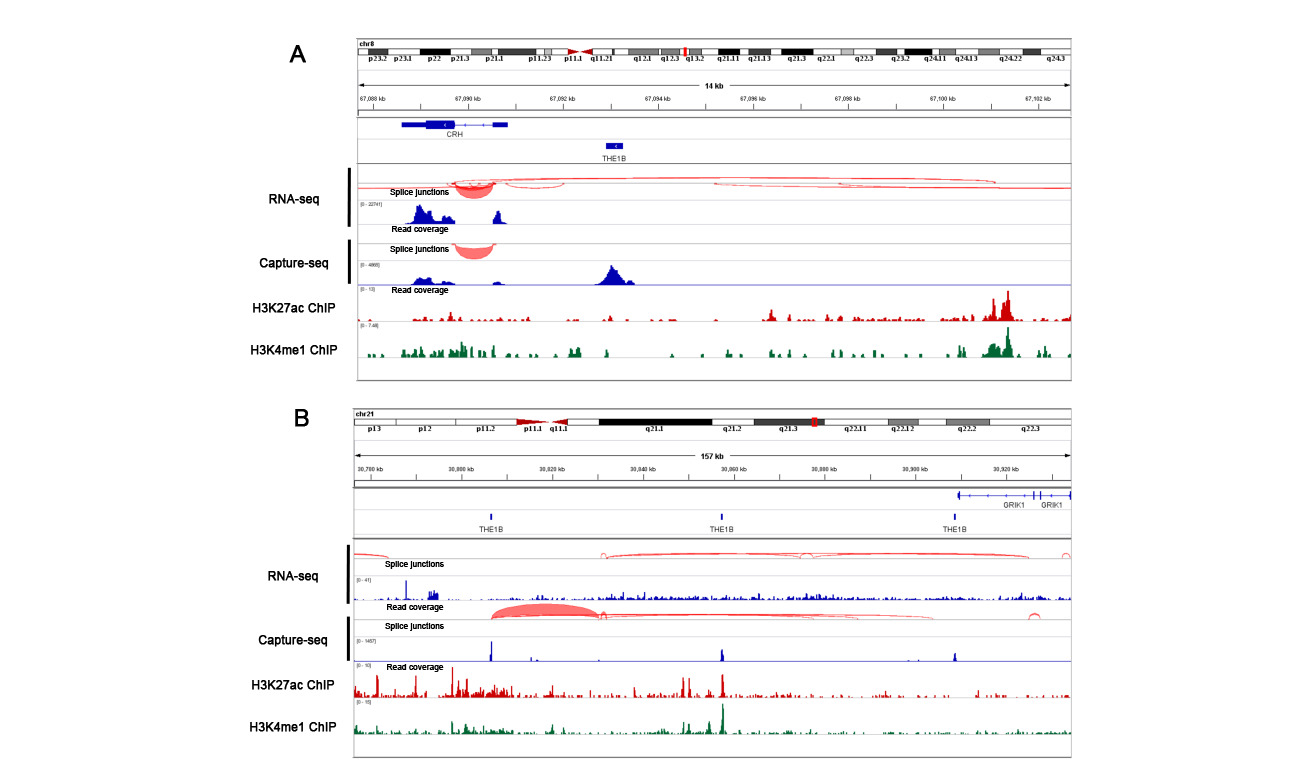

Supplement: S1 Fig — (A) Comparison of reads from human placental transcriptome pre-THE1B capture (RNA-seq) and post-THE1B capture (Capture-seq) to ChIP-seq reads for H3K27ac and H3K4me1 at the THE1B near CRH. This THE1B element lacks these chromatin marks, which are commonly associated with enhancers. (B) Comparison of reads from human placental transcriptome pre-THE1B capture (RNA-seq) and post-THE1B capture (Capture-seq) to ChIP-seq reads for H3K27ac and H3K4me1 at several THE1B elements near the GRIK1 gene. Enrichment of these chromatin marks may indicate enhancer activity. Raw data for A and B can be found at GEO accession number GSE118289. ChIP-seq, chromatin immunoprecipitation sequencing; GEO, Gene Expression Omnibus; H3K4me1, histone H3 lysine 4 monomethylation; H3K27ac, histone H3 lysine 27 acetylation; RNA-seq, RNA sequencing; THE1B, transposon-like human element 1B. (TIF) [file pbio.2006337.s003.tif]

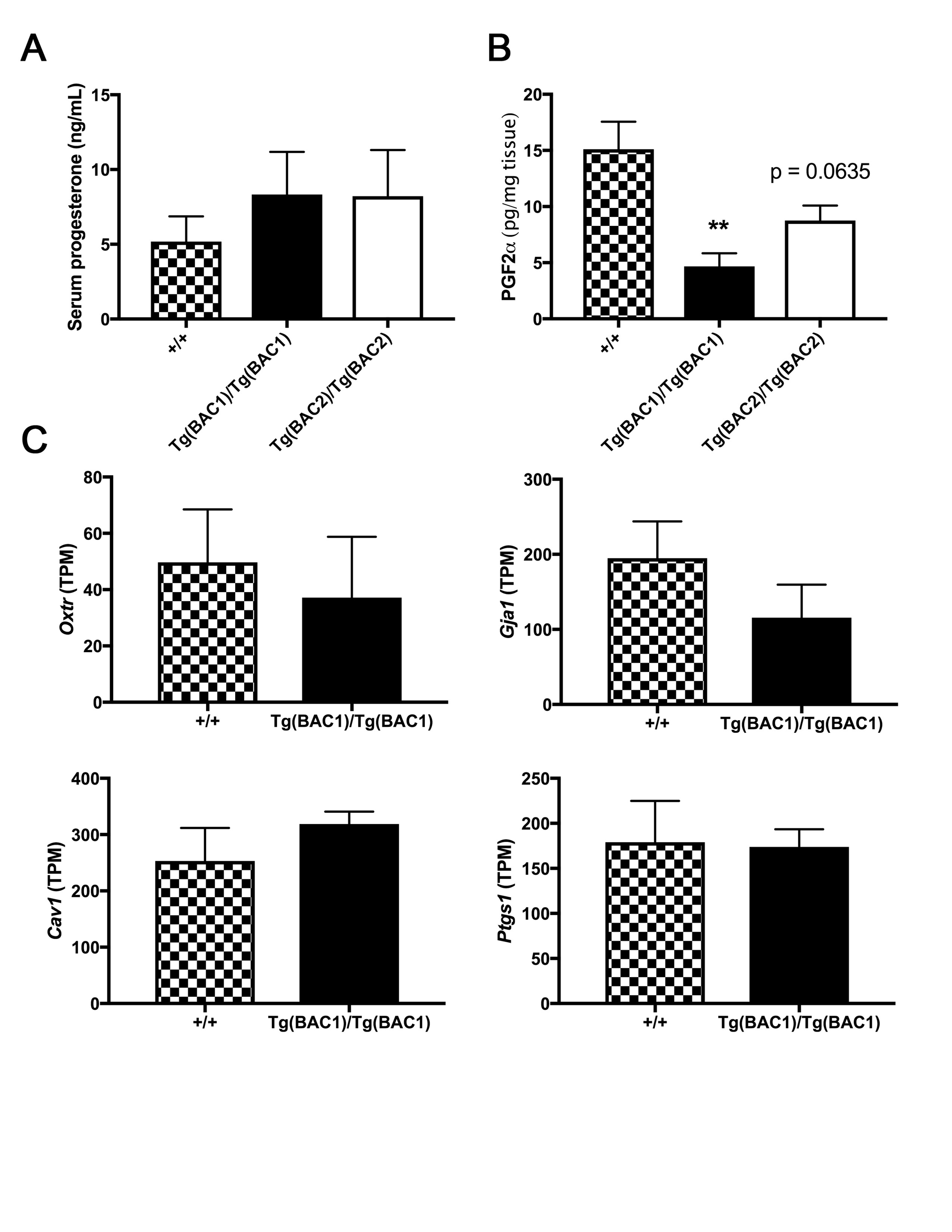

Supplement: S2 Fig — (A) Progesterone concentration in maternal serum at E18.5. (n = 5–10 per group, P = 0.6342 by one-way ANOVA. Error bars indicate the standard error of the mean.) (B) Prostaglandin F2α concentration in uterine tissue at E18.5 is significantly lower in Tg(BAC1) litters and approaches significance in Tg(BAC2) litters. (n = 7–11 per group, P = 0.0021 by one-way ANOVA. When compared to +/+, P = 0.0012 for Tg[BAC1]/Tg[BAC1] and P = 0.0635 for Tg[BAC2]/Tg[BAC2] by post hoc Dunnett’s multiple comparisons test. Error bars indicate the standard error of the mean.) (C) Expression of contractile-associated proteins in uterus of Tg(BAC1) and control animals measured by RNA-seq at E18.5. (n = 4 +/+, n = 3 Tg[BAC1]/Tg[BAC1]. All error bars indicate the standard error of the mean and were analyzed by unpaired two-tailed t-test. Oxtr, P = 0.6795. Gja1, P = 0.3005. Cav1, P = 0.4035. Ptgs1, P = 0.9313.) Ptgs2 expression was uniformly very low (TPM < 3.5 for all samples, P = 0.6485). Raw data can be found at GEO accession GSE118283. Numerical data for A, B, and C can be found in S1 Data. E18.5, embryonic day 18.5; GEO, Gene Expression Omnibus; RNA-seq, RNA sequencing; TPM, transcripts per million. (TIF) [file pbio.2006337.s004.tif]

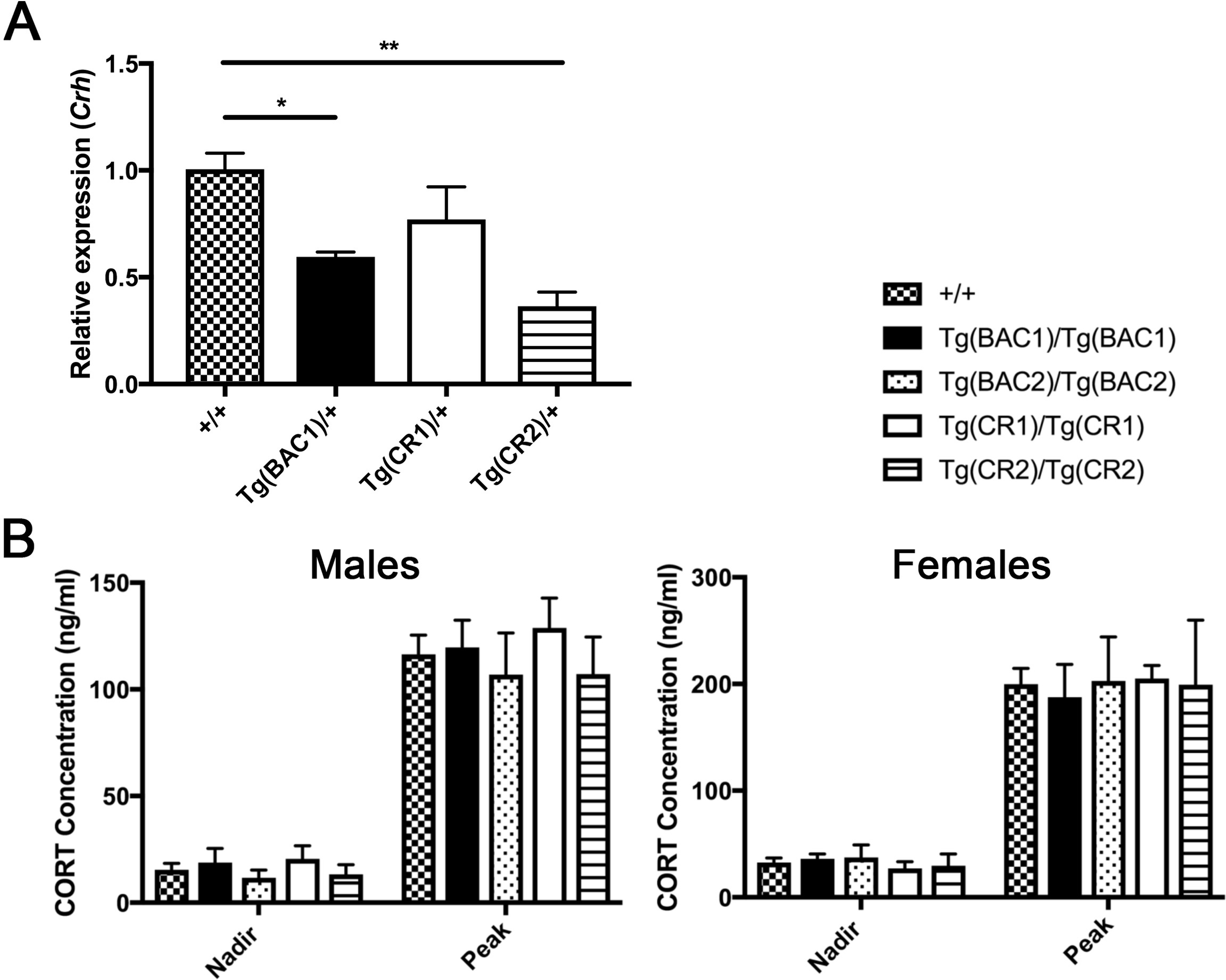

Supplement: S3 Fig — (A) Relative expression of mouse Crh in hypothalamus. Expression of Crh is down-regulated to compensate for addition of CRH expression in transgenic mouse hypothalamus. (n = 3 all groups, P = 0.0068 by one-way ANOVA. When compared to +/+, P = 0.0340 for Tg[BAC1]/+ and P = 0.0030 for Tg[CR2]/+ by post hoc Dunnett’s multiple comparisons test. Error bars indicate the standard error of the mean.) (B) Nadir and peak corticosterone (“CORT”) concentration in the serum of male (left) and female (right) transgenic mice. (n ≥ 3 all groups, P = 0.6568 for males and P = 0.9978 for females by two-way ANOVA, with genotype as the source of variation. Error bars indicate the standard error of the mean.) Numerical data for A and B can be found in S1 Data. (TIF) [file pbio.2006337.s005.tif]

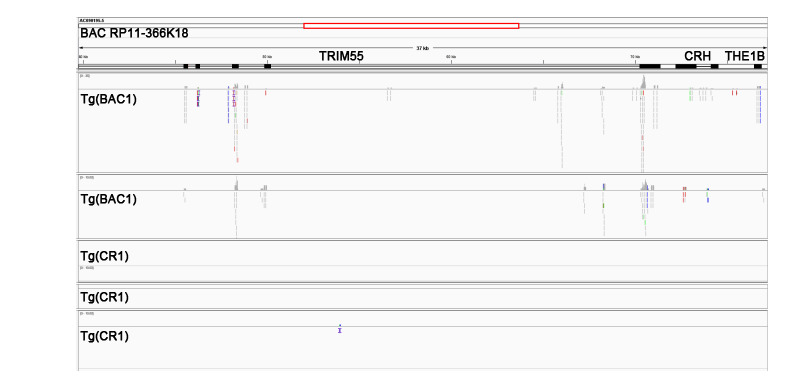

Supplement: S4 Fig — Gene expression from the human BAC in mouse placenta measured by paired-end RNA-seq. The only other protein-coding gene on the BAC, TRIM55, is located in a tail-to-tail orientation very close to CRH. Tg(BAC1) animals express TRIM55 and CRH in placenta, whereas Tg(CR1) animals do not. Red bar, highlighted region of BAC RP11-366K18 shown to scale. Raw data can be found at GEO accession GSE118283. BAC, bacterial artificial chromosome; GEO, Gene Expression Omnibus; RNA-seq, RNA sequencing; THE1B, transposon-like human element 1B. (TIF) [file pbio.2006337.s006.tif]

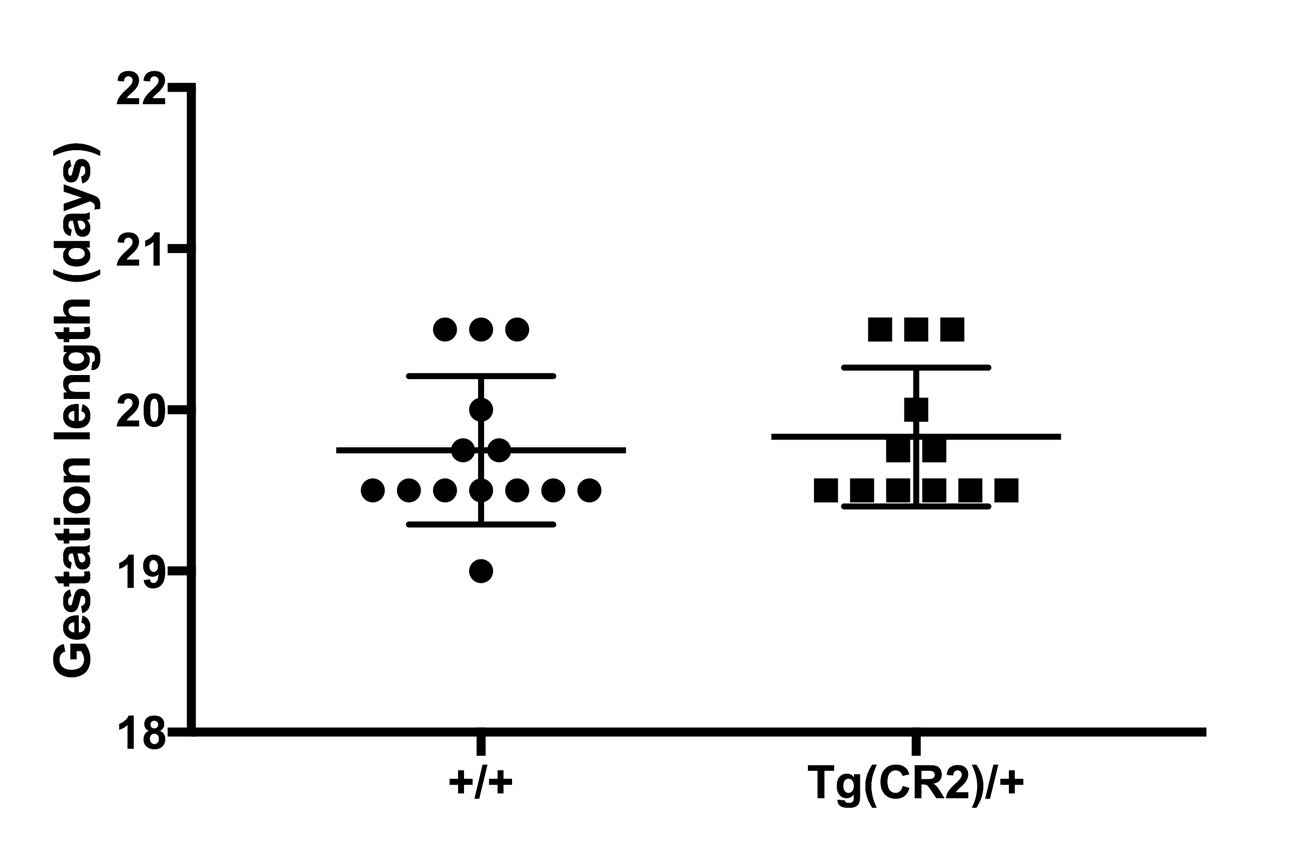

Supplement: S5 Fig — Gestation length of Tg(CR2)/+ and control litters. Although Tg(CR2) transgenic animals retain a small amount of placental CRH expression, litters receiving Tg(CR2) only from the father have no difference in gestation length when compared to wild-type control litters. (n = 14 +/+, n = 12 Tg[CR2]/+, P = 0.6397 by unpaired two-tailed t-test. Error bars indicate standard deviation.) Numerical data can be found in S1 Data. (TIF) [file pbio.2006337.s007.tif]
