# Supplementary material for: Anthropoid primate–specific retroviral element THE1B controls expression of CRH in placenta and alters gestation length
Source: PLoS Biol. 2018 Sep 19;16(9):e2006337. doi: 10.1371/journal.pbio.2006337 (PMC6166974; doi:10.1371/journal.pbio.2006337)
Supplement: S1 Table — Red text denotes predicted splice sites for anthropoid primate species. Primate genome sequences from UCSC Genome Browser, accessed February 27, 2018. CRH, corticotropin-releasing hormone; THE1B, transposon-like human element 1B. (DOCX) [file pbio.2006337.s008.docx]

| Species | Splice Donor |
| --- | --- |
| Human | AGTTCATAAAGGAAAGTAAGCAAAAATGTTGA |
| Chimpanzee | AGTTCATAAAGGAAAGTAAGCAAAAATGTTGA |
| Bonobo | AGTTCATAAAGGAAAGTAAGCAAAAATGTTGA |
| Gorilla | AGTTCATAAAGGAAAGTAAGCAAAAATGTTGA |
| Orangutan | AGGTCATAAAAGAAAGTAAGCAAAAATGTTGA |
| Gibbon | AGGTCATAAGGGAAAGTAAGCAAAAATGTTGA |
| Rhesus macaque | AGGTCATAAAGGAAAGTAAGCAAAAATGTTGA |
| Crab-eating macaque | AGGTCATAAAGGAAAGTAAGCAAAAATGTTGA |
| Baboon | AGGTCATAAAGGAAAGTAAGCAAAAATGTTGA |
| Green monkey | AGGTCATAAAGGAAAGTAAGCAAAAATGTTGA |
| Golden snub-nosed monkey | AGGTTATAAAGGAAAGTAAGCAAAAATGTTGA |
| Marmoset | AGGTCATAAAGGAGAGTAAGCCAAAATGTTGA |
| Squirrel monkey | AGGTCATAAAGGAGAGTGAGCCAAAATGTTGA |
| Tarsier | GGGTCATACAGGAAATGAAGCAGACATATCAA |
| Mouse lemur | GGGTCATAAAGTAAAGTAAGCAAAAACATTGA |
| Bushbaby | GGGTCATAAAGGAAAGTAAAGTAAAATATTGA |

| Species | Splice Acceptor |
| --- | --- |
| Human | CCCTCGTTCCTTGGCAGGGCCCTATGATTTAT |
| Chimpanzee | CCCTCGTTCCTTGGCAGGGCCCTATGATTTAT |
| Bonobo | CCCTCGTTCCTTGGCAGGGCCCTATGATTTAT |
| Gorilla | CCCTCGTTCCTTGGCAGGGCCCTATGATTTAT |
| Orangutan | CCCTCGTTCCTTGGCAGGGCCCTATGATTTAT |
| Gibbon | CCCTCGTTCCTTGGCAGGGCCCTATGATTTAT |
| Rhesus macaque | CCCTCGTTCCTTGGCAGGGCCCTATGATTTAT |
| Crab-eating macaque | CCCTCGTTCCTTGGCAGGGCCCTATGATTTAT |
| Baboon | CCCTCGTTCCTTGGCAGGGCCCTATGATTTAT |
| Green monkey | CCCTCGTTCCTTGGCAGGGCCCTATGATTTAT |
| Golden snub-nosed monkey | CCCTCGTTCCTTGGCAGGGCCCTATGATTTAT |
| Marmoset | CCCTCGCTCCTTGGCAGGGCCCTATGATTTAT |
| Squirrel monkey | CCCTCGCTCCTTGGCAGGGCCCTATGATTTAT |
| Tarsier | CCCTCGCTCCTTGGCAGGGCCCTATGATTTAT |
| Mouse lemur | CCCTCGCTCCTTGGCAGG-CCCTATGATTTAT |
| Bushbaby | CCCTCGCTCTTTGGCAGGGCCCTATGATTTAT |
